# Large *Drosophila* germline piRNA clusters are evolutionarily labile and dispensable for transposon regulation

**DOI:** 10.1016/j.molcel.2021.07.011

**Published:** 2021-10-07

**Authors:** Daniel Gebert, Lena K. Neubert, Catrin Lloyd, Jinghua Gui, Ruth Lehmann, Felipe Karam Teixeira

**Affiliations:** 1 Department of Genetics, University of Cambridge, Cambridge CB2 3EH, UK; 2 Howard Hughes Medical Institute (HHMI) and Kimmel Center for Biology and Medicine of the Skirball Institute, Department of Cell Biology, New York University School of Medicine, New York, NY 10016, USA

**Keywords:** germ cells, transposable elements, gene silencing, heterochromatin, chromosome inversions, position-effect variegation, gypsy12, flamenco, sterility, fruit fly

## Abstract

PIWI proteins and their guiding Piwi-interacting small RNAs (piRNAs) are crucial for fertility and transposon defense in the animal germline. In most species, the majority of piRNAs are produced from distinct large genomic loci, called piRNA clusters. It is assumed that germline-expressed piRNA clusters, particularly in *Drosophila*, act as principal regulators to control transposons dispersed across the genome. Here, using synteny analysis, we show that large clusters are evolutionarily labile, arise at loci characterized by recurrent chromosomal rearrangements, and are mostly species-specific across the *Drosophila* genus. By engineering chromosomal deletions in *D. melanogaster*, we demonstrate that the three largest germline clusters, which account for the accumulation of >40% of all transposon-targeting piRNAs in ovaries, are neither required for fertility nor for transposon regulation in *trans*. We provide further evidence that dispersed elements, rather than the regulatory action of large *Drosophila* germline clusters in *trans*, may be central for transposon defense.

## Highlights

• Fly piRNA clusters are evolutionarily short lived and arise at unstable genomic loci
• Flies lacking the three major germline piRNA clusters are viable and fertile
• Despite loss of >40% of transposon-derived piRNAs, transposons are not activated
• Rather than by large clusters, regulation may be achieved in *cis* by dispersed elements

## Graphical abstract

**Figure:**
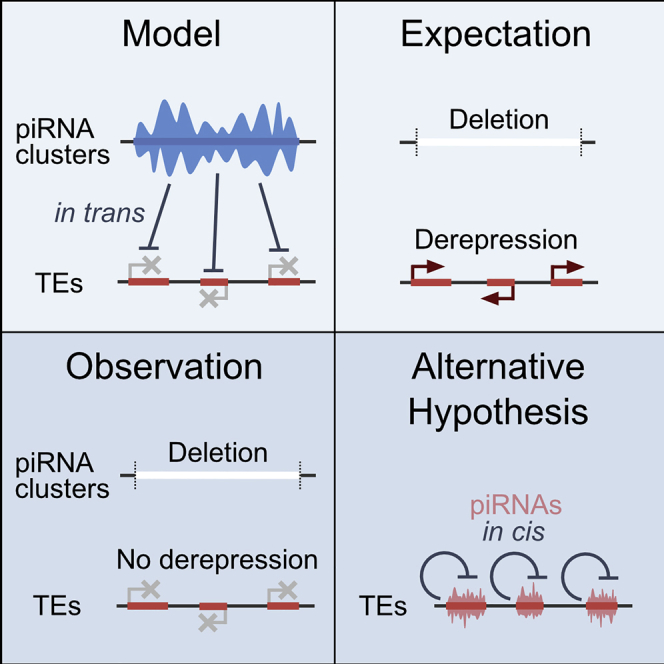

## Teaser

Large piRNA clusters are considered principal regulators of transposon activity in the germline of animals. Gebert et al. challenge this model by showing that these genomic loci appear and disappear rapidly in *Drosophila* evolution and that their engineered deletion has no effect on fertility or transposon activity in fruit flies.

## Introduction

In the germline of animals, the piwi-interacting small RNA (piRNA) pathway is an essential defense mechanism against transposable elements (TEs) (Aravin et al., 2007; Brennecke et al., 2007; Czech et al., 2018). PIWI proteins of the Argonaute family and their associated 23–30 nt long small RNAs form the cores of effector protein complexes that recognize RNA transcripts through sequence complementarity to initiate silencing at both the transcriptional (Aravin et al., 2008; Wang and Elgin, 2011) and post-transcriptional levels (Brennecke et al., 2007; Czech et al., 2018; Gunawardane et al., 2007). Mutations affecting the piRNA pathway consistently induce TE upregulation, which in turn is thought to underlie the germline developmental defects that ultimately lead to animal sterility (Carmell et al., 2007; Cox et al., 1998; Klattenhoff et al., 2007; Schüpbach and Wieschaus, 1991).

Many components of the piRNA pathway are evolutionarily conserved across the animal kingdom, with PIWI protein homologs identified in genomes from cnidaria to humans (Lim et al., 2014). Genetic screens have been extensively used to identify piRNA pathway protein components in model organisms and molecular, biochemical, and developmental analyses have helped dissect the role of new components on small RNA biogenesis and silencing, leading to an increasingly refined molecular understanding of the pathway (Czech et al., 2018). piRNA biogenesis relies on the activity of conserved endonucleases and PIWI proteins, but unlike other small RNA pathways, it operates independently of Dicer proteins (Brennecke et al., 2007; Gunawardane et al., 2007). In the germline in flies and mammals, the abundance of PIWI-piRNA complexes is further magnified by the ping-pong cycle, an amplification loop based on the slicing activity of cytoplasmic PIWI proteins. Finally, silencing is mediated by piRNA-loaded effector complexes that either induce target transcript slicing by PIWI-mediated cleavage or recruit additional complexes that direct chromatin modification at the target loci (Czech et al., 2018).

In many species, the majority of piRNAs are derived from the processing of non-coding transcripts that originate from a small number of large genomic loci called piRNA clusters (Aravin et al., 2006, 2007; Brennecke et al., 2007; Chirn et al., 2015; Gebert et al., 2019; Girard et al., 2006). In *Drosophila*, these piRNA-producing loci, which can be up to hundreds of thousands of base pairs long, are densely populated with TEs. Although a few of these loci are uni-directionally transcribed (uni-strand clusters), the vast majority of the ∼100 piRNA clusters active in the *Drosophila* germline are transcribed from both DNA strands (dual-strand clusters). This is achieved by non-canonical convergent Pol II transcription, which is mediated by specialized machinery and is set off by chromatin marks rather than DNA sequence motifs. In this case, resulting RNA precursors are not spliced or polyadenylated (Andersen et al., 2017; Brennecke et al., 2007; Klattenhoff et al., 2009; Mohn et al., 2014). Regardless of the type of transcription used, a few highly expressed piRNA clusters located at the euchromatic-pericentromeric borders produce the bulk of piRNAs in gonads and therefore were proposed to act as principal regulators of transposon activity (Brennecke et al., 2007; Czech et al., 2018; Malone and Hannon, 2009). Furthermore, the currently favored model postulates that piRNA clusters acquire the ability to regulate new and active TEs through random integration, providing immunity against all cognate transposon copies throughout the genome and building up a memory immune system (Khurana et al., 2011). In this context, it is hypothesized that piRNA clusters are adaptive loci that play a key role in an evolutionary arms race between host genomes and TEs (Levine and Malik, 2011; Malone and Hannon, 2009).

In contrast to many protein components of the piRNA pathway, which although conserved were shown to be rapidly evolving under positive selection in *Drosophila* (Obbard et al., 2009; Parhad et al., 2017; Simkin et al., 2013), the conservation of piRNA-producing loci has not yet been thoroughly studied. To date, a few *D. melanogaster* piRNA clusters have been subject to evolutionary analysis, revealing that the conservation of both the somatic *flamenco* locus and the germline *42AB* piRNA cluster is restricted to a few closely related species separated by 2–7 million years (My) (Chirn et al., 2015; Malone et al., 2009). More extensive analyses have been performed in mammals, indicating that although piRNA-producing loci are generally poorly conserved compared with protein-coding genes, a few large pachytene piRNA clusters are conserved over extended evolutionary times (Assis and Kondrashov, 2009; Chirn et al., 2015; Gebert et al., 2019; Özata et al., 2020). Despite this, only a subset of such evolutionary conserved pachytene piRNA loci were shown to be important for mouse fertility (Wu et al., 2020).

Here, we took advantage of nanopore-sequenced genome assemblies (Miller et al., 2018), embryonic small RNA data (Mohammed et al., 2018), and synteny comparisons to analyze approximately 70 My of genome evolution in the *Drosophila* genus. Using synteny analysis, we show that large germline piRNA clusters, and piRNA-producing loci in general, are extremely labile and not evolutionarily conserved. Moreover, we found that piRNA clusters arise at loci that are characterized by their association with recurrent chromosomal rearrangements through *Drosophila* evolution, suggesting that unstable genomic regions are prone to piRNA cluster genesis. To directly test the role of large germline piRNA-producing loci in TE regulation and genome stability, we generated genomic deletions of the three major germline piRNA clusters in *D. melanogaster*. Our genetic, molecular, and developmental analyses indicate that germline piRNA clusters are dispensable for TE and gene regulation and have no impact on transposon mobilization in *trans* or on fertility. Altogether, we provide evidence that large germline piRNA clusters in *Drosophila* are not only evolutionary labile but also mostly dispensable for endogenous transposon regulation in flies.

## Results

### Evolution of germline-expressed piRNA clusters in the *Drosophila* genus

**Figure 1:**
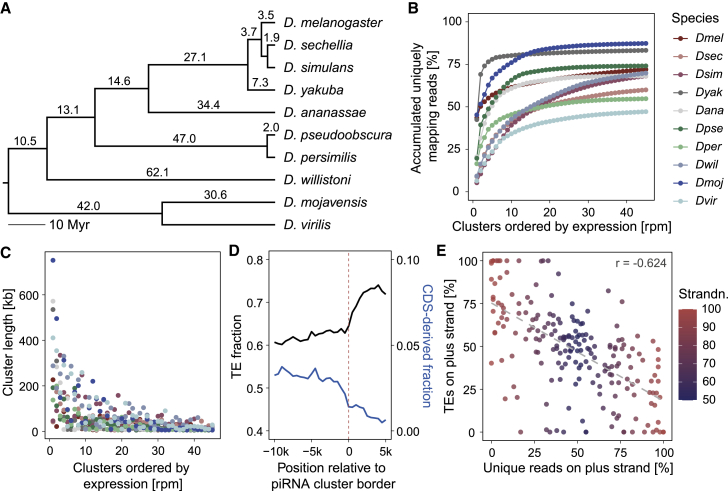
Identification of germline piRNA clusters in ten *Drosophila* species (A) Phylogenetic tree of *Drosophila* species used in this study. Divergence times as described in Thomas and Hahn (2017). Outgroup species (*Musca domestica*) was omitted. (B) Accumulated uniquely mapping reads from germline piRNA clusters ordered by decreasing expression for each species. (C) Length of germline piRNA clusters ordered by decreasing expression for each species. (D) Average fraction of base pairs overlapping with TE and coding sequence (CDS)-derived annotations (500 bp windows) at the borders (dashed line) of the top 20 largest piRNA clusters of each species, including 10 kb flanking regions and 5 kb of internal cluster regions. (E) Distribution of TE insertions on the plus strand and uniquely mapping reads on the plus strand for the top 20 largest germline piRNA clusters of each species. Color gradient represents the strandedness in percentage of reads on the major strand. Dashed line depicts linear regression. r, Pearson correlation coefficient. See also Figures S1–S3.

To study the evolutionary dynamics of germline piRNA clusters through relatively short periods of time, we focused on ten species closely related to *D. melanogaster*, representing ∼73 My of evolution within the *Drosophila* genus (Figure 1A). Unbiased identification of germline-expressed piRNA clusters was conducted by combining previously annotated genome drafts (Adams et al., 2000; Drosophila 12 Genomes Consortium et al., 2007; Hoskins et al., 2015), recently published Nanopore long-read-based genome assemblies (Miller et al., 2018; Shah et al., 2019), and species-specific, high-throughput small RNA sequencing (RNA-seq) data obtained from mixed-aged embryos, which contain maternally deposited small RNAs generated during oogenesis as well as small RNAs zygotically produced during embryogenesis (see STAR Methods; Barckmann et al., 2015; Mohammed et al., 2018). After the removal of known microRNAs (miRNAs), small interfering RNAs (siRNAs), other non-coding and structured RNAs, as well as gene and pseudogene sequence from small RNA data (Figure S1A), the distribution and density of uniquely mapping 23–29 nt piRNAs across the genomes were used to identify piRNA-producing loci in each species (Figures S1B–S1D; Table S1). As previously observed in *D. melanogaster* (Brennecke et al., 2007), the majority of unique piRNAs were found to be clustered around a small number of large loci in all analyzed species (Figures 1B and 1C). These loci were enriched for repetitive elements and depleted for gene coding sequences while surrounded by regions characterized by lower repeat content and higher gene content (Figure 1D), which is reminiscent of the piRNA clusters described in *D. melanogaster* (Brennecke et al., 2007). Most identified clusters were expressed from both strands (i.e., dual-strand clusters; Figure 1E; Figure S2A), showing strong ping-pong signatures when either unique or all mapped sequences were considered (Figures S2B, S2C, and S3A), a hallmark of germline piRNA biogenesis (Malone et al., 2009). On average, uni-strand clusters (i.e., >95% of uniquely mapping piRNAs generated from one strand) were smaller in size (Figure S2D) and were disproportionately enriched for TEs oriented in the antisense direction to the accumulation of piRNAs (Figure 1E; Figure S3B; Table S1). In summary, these results indicate that similar to what was observed in *D. melanogaster*, large germline piRNA clusters are responsible for the production of most unique piRNAs in each analyzed species.

**Figure 2:**
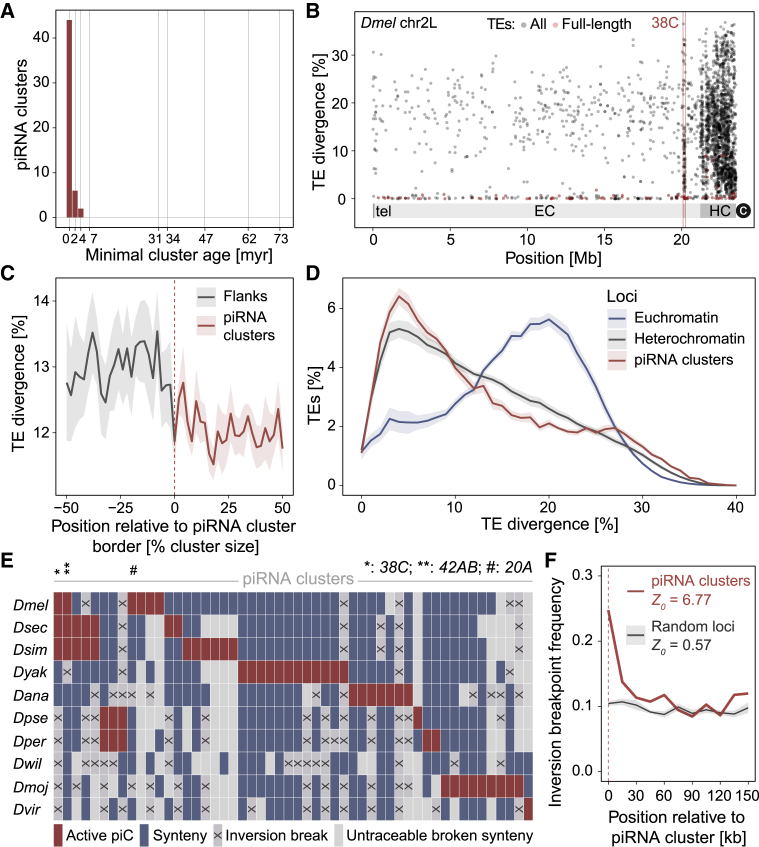
Evolution of germline piRNA clusters in the *Drosophila* genus (A) Age distribution of the top 20 largest germline piRNA clusters of each species with identified syntenies. (B) Percentage of sequence divergence from consensus for individual TE copies across the *D. melanogaster* chromosome 2L. EC, euchromatin; HC, heterochromatin; tel, telomere; c, centromere. The germline piRNA cluster *38C* is highlighted in red. Full-length TE copies are shown in pink. (C) Averaged TE sequence divergence at the borders (dashed line) of the top 20 largest germline piRNA clusters of each species, including halves (50% length) or internal cluster regions and flanking regions of corresponding lengths (−50%). (D) Average TE sequence divergence distribution of copies found within the top 20 largest germline piRNA clusters of each species, and in euchromatic and heterochromatic genomic regions. Standard errors are displayed in fainted color areas. (E) Synteny analysis for 61 germline piRNA clusters with synteny in at least two species. (F) Frequency of inversion breakpoint events (15 kb windows) observed in the genome of ten *Drosophila* species at the flanks of germline piRNA clusters (red line) and at random genomic loci (black line). The x axis represents the distance in kilobases to the piRNA cluster border or random locus border (z = 6.77 corresponds to p < 0.00001; z = 0.57 corresponds to p = 0.284339). See also Figures S4 and S5.

Because of the repetitive nature of piRNA cluster sequences, we took advantage of gene-rich flanking regions and performed synteny analysis to follow cluster evolution through the *Drosophila* genus. Focusing on the 20 top clusters of each species, which account for 45%–85% of all unique piRNAs in the respective embryonic small RNA libraries (Figure 1B), we were able to recapitulate the evolutionary history for 61 of them. From these, 45 clusters were shown to be species-specific, 16 were shown to be conserved through >∼1.9 My, but none of them was conserved through >∼7.3 My (Figure 2A). In agreement with this, sequence divergence analysis indicated that piRNA clusters were enriched for low divergent (likely “young”) transposon insertions compared with nearby regions (Figures 2B and 2C; Figure S4A). Moreover, global analysis revealed a specific TE sequence divergence for piRNA clusters that is fundamentally different from euchromatin and closer to heterochromatin, although piRNA clusters showed a stronger enrichment for less divergent TE insertions compared with heterochromatin as well (Figure 2D; Figures S4B and S4C). In conclusion, and against the prevalent view that piRNA clusters are particularly enriched in TE relics and considered as TE “graveyards” (Malone and Hannon, 2009), we observed that large germline-expressed piRNA clusters are enriched in rather low divergent (“younger”) repeats. Moreover, our analysis on piRNA cluster location turnover revealed that despite being present in all analyzed species, individual piRNA clusters are not preserved through long evolutionary periods.

Remarkably, our evolutionary analysis indicated that genome synteny is recurrently and specifically interrupted at loci permissive to the emergence of germline piRNA clusters, regardless of the presence or absence of clusters or TEs (Figure 2E; Figure S5). Indeed, analysis of the distribution of chromosomal rearrangement events occurring through evolution revealed an increased frequency of synteny breaks coinciding with narrow genome windows (i.e., in between pair of flanking genes) that are apparently tolerant to the appearance of piRNA clusters (Figure 2F). Therefore, we concluded that genomic loci permissive to the emergence of piRNA clusters are disproportionately involved in chromosomal rearrangements in comparison with flanking regions and the genome overall.

### Generation of site-specific genomic deletions encompassing the major germline piRNA clusters in *D. melanogaster*

**Figure 3:**
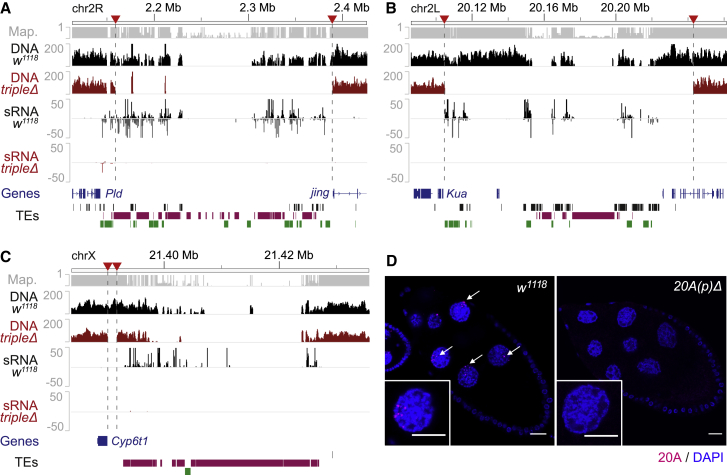
Chromosomal deletions spanning the germline piRNA clusters *42AB*, *38C*, and *20A* (A) Comparison of *w1118* control strain and *tripleΔ* mutant at *42AB* locus. Genome Browser tracks with density plots for mappability (Map.), uniquely mapping DNA-seq reads (DNA), and uniquely mapping small RNA-seq reads (sRNA). Annotation is at the bottom: genes (blue), DNA transposons (black), LTR retrotransposons (purple), and non-LTR retrotransposons (green). Red arrows indicate FRT sites used to generate cluster deletions. (B) Comparison of *w1118* control strain and *tripleΔ* mutant at *38C* locus. (C) Comparison of *w1118* control strain and *tripleΔ* mutant at *20A* locus. (D) Representative confocal projection of RNA-FISH signal for *20A* sense probes (red) in *w1118* and *20A(p)Δ* mutant ovaries. Insets depict projections of representative nurse cell nuclei for the same genotypes. DAPI (DNA, blue). Scale bars, 20 μM. See also Figure S6.

The large abundance of small RNAs originating from piRNA clusters (Figure 1B) as well as studies focused on the analysis of the somatic piRNA-producing *flamenco* locus, which regulates the expression of *Gypsy* elements in somatic tissues, led to a model in which large piRNA clusters are principal regulators of transposon activity (Brennecke et al., 2007; Malone and Hannon, 2009). However, on the basis of our synteny analysis, we observed that large germline piRNA clusters are evolutionarily labile and originate at genomic regions involved in recurrent chromosomal rearrangements through evolution. To directly test the role of large germline piRNA clusters on transposon regulation and genome stability, we elected to generate site-specific chromosome deletions to disrupt the three major germline piRNA clusters in *D. melanogaster* (namely, *42AB*, *20A*, and *38C*). To do so, we used the FRT-based strategy established by Golic and Golic (1996) and further developed by the chromosomal deletion projects (Ryder et al., 2004; Thibault et al., 2004). First, we identified publicly available, FRT-bearing P element transgenic insertions generated by the *Drosophila* Gene Disruption Project (Bellen et al., 2011; Ryder et al., 2004) that are located at or in the vicinity of germline piRNA cluster extremities (Figures 3A–3C; Table S2). Then, using FLP-mediated recombination between two FRT sites located at opposite extremities of each cluster, we successfully generated independent chromosomal deletions encompassing the two major dual-strand germline piRNA clusters in *D. melanogaster: 42AB* (also known as cluster 1) and *38C* (originally named cluster 5 and cluster 27; Brennecke et al., 2007). DNA sequencing (DNA-seq) comparisons conducted on DNA extracted from control and homozygous mutant flies carrying the deletions confirmed that the clusters were specifically removed while flanking sequences were preserved (Figures 3A and 3B): the *42AB* deletion (*42ABΔ*; Figure 3A) removed ∼230 kb of the respective cluster (95%), while the *38C* deletion (*38CΔ*; Figure 3B) was confirmed to be ∼138 kb long and to encompass the totality of clusters 5 and 27. Moreover, small RNA-seq analyses conducted with RNA extracted from ovaries of control and homozygous mutant flies confirmed that cluster deletions led to the complete loss of unique piRNAs from the respective regions (Figures 3A and 3B).

Located upstream of the *flamenco* locus at the pericentromeric border of the X chromosome, cluster *20A* is the major uni-strand germline-expressed piRNA cluster found in the *D. melanogaster* genome (Brennecke et al., 2007). To generate fly lines lacking a functional *20A* cluster, we used two strategies. First, we took advantage of previously established large chromosomal deletions encompassing *20A*. By means of genetic crosses using genomic duplications to overcome male lethality induced by X chromosome deletions (Cook et al., 2010), we were able to combine a pair of large deletions that exclusively spanned the *20A* locus (Figure S6A). DNA-qPCR was used to validate the absence of *20A* DNA in *trans*-heterozygous mutant flies (data not shown), and small RNA-seq analyses confirmed that production of small RNAs from *20A* was eliminated, while piRNAs from the nearby *flamenco* locus were unaffected (Figures S6A and S6B).

In parallel, and mainly because of the difficulties of working with large deletions on the X chromosome, we identified a pair of FRT-bearing P element transgenic insertions that flanked what we hypothesized to be the transcriptional start site (TSS) of the *20A* locus. The TSS was located ∼1 kb upstream of the original cluster coordinates and was identified through the analysis of cap analysis of gene expression sequencing (CAGE-seq) data generated by the modENCODE consortium on adult ovaries (Figure S6B; SRR488282; Hoskins et al., 2011). Using FLP-mediated recombination between the two FRT sites, we generated a 1.6-kb-long deletion upstream of *20A* that specifically eliminated the putative TSS sequence (Figure 3C). This deletion, named hereafter *20A(p)Δ*, did not overlap with the original *20A* coordinates. Small RNA-seq analysis conducted with ovaries of *20A(p)Δ* homozygous mutants demonstrated that the 1.6-kb-long deletion was sufficient to eliminate the production of small RNAs from the 39.7-kb-long *20A* cluster to the same extent as we observed with the large overlapping deletions. Moreover, the accumulation of small RNAs from the neighboring *flamenco* locus was unaffected in 2*0A(p)Δ* homozygotes, confirming the specificity of the deletion (Figure S6A). To investigate whether the loss of piRNAs observed in *20AΔ(p)* homozygous mutants was due to the absence of transcriptional activity or to the disruption of piRNA processing, we performed RNA fluorescent *in situ* hybridization (FISH) using *20A* probes that did not overlap with the *20A(p)Δ* deletion. In control ovaries, many nuclear foci, likely transcription sites, were observed in germline nurse cells, confirming the germline specificity of *20A* (Figure 3D). In contrast, we did not detect any nuclear RNA focus in nurse cells of *20A(p)Δ* homozygous mutants, demonstrating that cluster *20A* transcription was eliminated by the deletion of the 1.6-kb-long *20A(p)Δ* fragment containing the putative upstream TSS. In conclusion, we were able to generate viable homozygous fly lines carrying site-specific chromosomal deletions that affect the major germline clusters and eliminate the piRNA production from the respective loci.

### Mutants disrupting major germline piRNA clusters are viable and fertile

**Figure 4:**
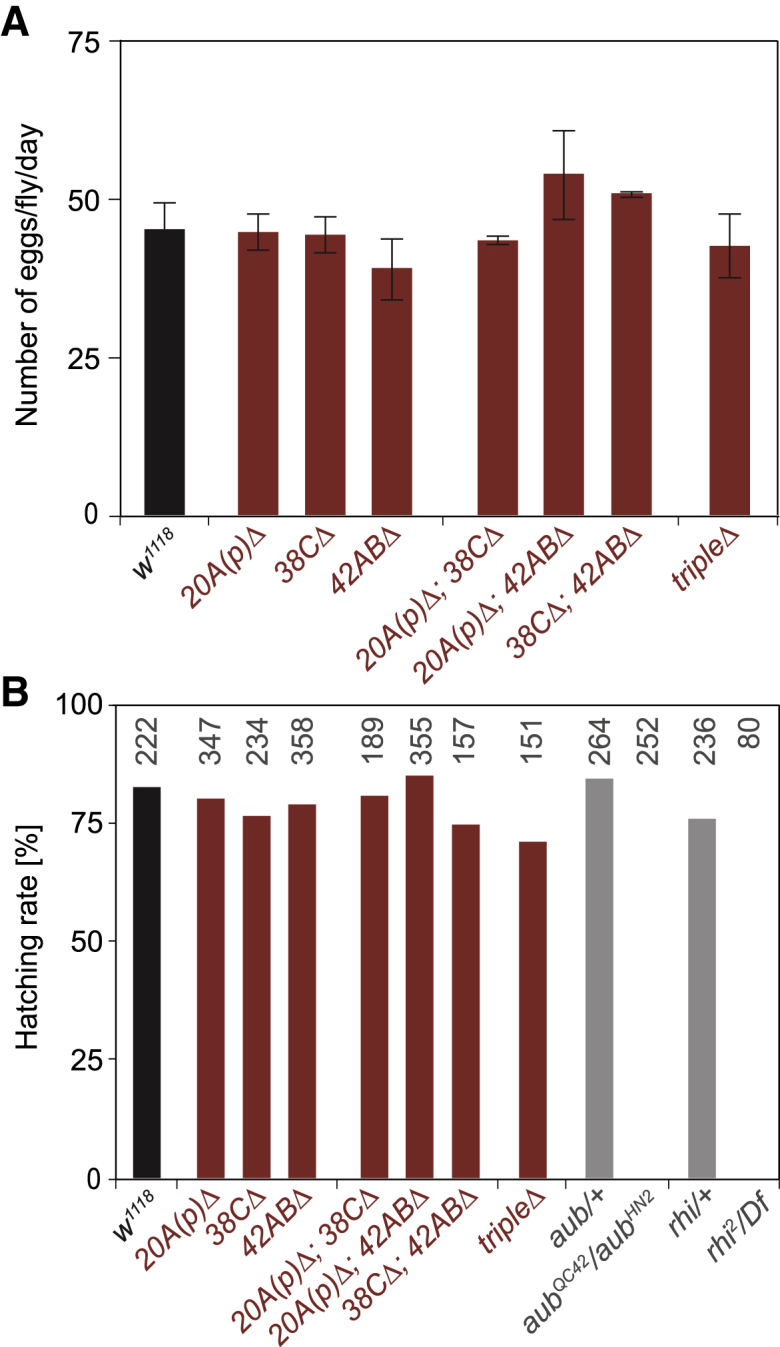
Female fertility test for piRNA cluster deletions (A) Number of eggs laid per fly per day in *w1118* flies and cluster deletion mutants. (B) Hatching rate of laid eggs for *w1118* flies, cluster deletion mutants and *aubergine* (*aub*) and *rhino* (*rhi*) heterozygotes and homozygous mutants. Numbers indicate the total number of eggs analyzed.

Initially described for their role in germline development, the protein components involved in the piRNA pathway were shown to be required for fertility in animals (Carmell et al., 2007; Cox et al., 1998; Klattenhoff et al., 2007; Schüpbach and Wieschaus, 1991). In *Drosophila*, mutations affecting germline-specific components of the pathway lead to stereotypical sterility phenotypes, which are characterized by viable homozygous mutant females that lay eggs that do not hatch. This phenotype has been extensively used to identify new components of the pathway and is due, at least in part, to the activation of the germline DNA damage checkpoint during oogenesis with consequential disruption of the egg dorsoventral axial polarity establishment and impairment of nuclear migration during early embryo development (Klattenhoff et al., 2007; Schüpbach and Wieschaus, 1991). To determine whether the disruption of large germline piRNA clusters caused germline developmental defects or fertility problems, we first characterized homozygous mutant females for egg-laying capacity. Our results indicated that deletion of germline piRNA clusters, individually or in combination, do not affect egg-laying output compared with control flies (Figure 4A). Therefore, we investigated the hatchability of eggs laid by mutants affecting germline-expressed piRNA clusters. We observed that eggs produced by single-, double-, or triple-mutant mothers hatched at the same frequency as controls (Figure 4B). This is in sharp contrast to what was observed for the protein components of the germline piRNA pathway—*aubergine* (*aub*) and *rhino* (*rhi*) mutants—for which none of the eggs laid by homozygous mutants hatched (Figure 4B; Klattenhoff et al., 2007, 2009). Therefore, our results indicate that in contrast to the genes encoding for the protein components of the germline piRNA pathway, the major germline piRNA clusters are not required for fertility, even when the three major clusters are simultaneously disrupted. Moreover, we did not observe other somatic or germline phenotypes in piRNA cluster mutants (data not shown), even when mutations were maintained in homozygosity for many generations.

### Mutants disrupting major germline piRNA clusters lead to a strong reduction in total piRNA accumulation

**Figure 5:**
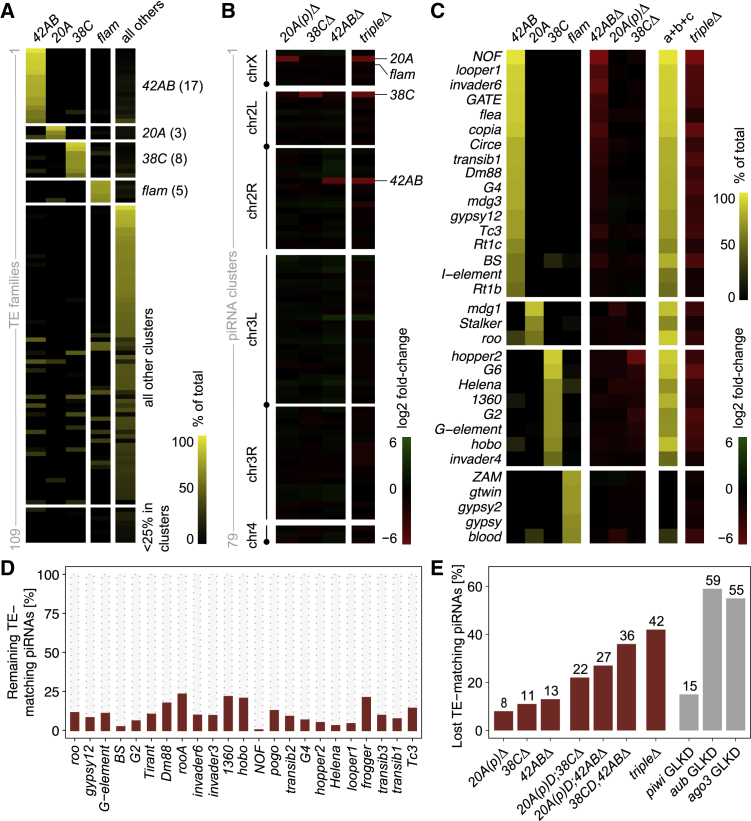
Effects of germline piRNA cluster deletions on piRNA accumulation (A) Distribution of piRNA reads (percentage of unique mappers) matching each TE family and piRNA clusters (*42AB*, *20A*, *38C*, *flamenco* [*flam*], and all the remaining clusters) in *w1118* ovaries. Numbers in brackets represent the numbers of TE families for which >50% of unique piRNA reads map to *42AB*, *20A*, *38C*, *flam*, or all remaining clusters. (B) Changes in the accumulation of unique piRNA reads (log_2_ fold change of unique mappers) matching piRNA clusters > 10 kb in mutant ovaries in comparison with *w1118* control (red-green gradient). (C) Distribution of piRNA reads (percentage of unique mappers) matching each TE family and piRNA clusters (*42AB*, *20A*, *38C*, and *flam*) in *w1118* ovaries (yellow gradient) and the log_2_ fold change of TE-matching piRNA reads (unique mappers) for each family in mutant ovaries in comparison with *w1118* control (red-green gradient). Column “a+b+c” represents the sum of percentages of unique TE-matching piRNA reads per TE family for clusters *42AB*, *20A*, and *38C* in *w1118* ovaries (yellow gradient). Pearson correlation coefficients (r) between percentage of unique mappers in *w1118* and log_2_ fold change in mutants: −0.750 (*42ABΔ*, p < 0.0001), −0.549 (*20A(p)Δ*, p = 0.0009), −0.884 (*38CΔ*, p < 0.0001), and −0.650 (*tripleΔ*, p < 0.0001). (D) Percentage of TE-matching piRNA reads (all mappers) per TE family for families with <25% of normalized piRNAs in triple-mutant ovaries compared to *w1118* control. (E) Percentage of lost TE-matching piRNA reads (all mappers) in piRNA cluster mutant ovaries and germline knockdowns for *piwi*, *aub*, and *ago3* (Olivieri et al., 2012; Senti et al., 2015) compared with controls. See also Figure S7.

Given the lack of noticeable phenotypic changes in the cluster mutants, we examined the effect of germline clusters disruption on piRNA accumulation. First, we used small RNA-seq data from *w*^*1118*^ ovaries and genomic analysis to better characterize the germline piRNA clusters in terms of their specificity, piRNA-producing capacity, and relationship to TE families. Although redundancy between multiple loci was observed for some TE families, the major germline piRNA clusters were predicted to be individually responsible for sustaining piRNA production of defined and non-overlapping sets of TE families. Indeed, the *42AB* locus was expected to be the major source of uniquely mapping piRNAs (>50%) for 17 transposon families, while another three TE families were strongly associated with the *20A* locus and additional eight TE families with the *38C* locus (Figure 5A). This is similar to what was observed for the somatic *flamenco* locus, which is the main source of uniquely mapping piRNAs for five TE families (Table S3). The bias in the relationship between clusters and TE families with regard to uniquely mapping piRNAs was also reflected in the noticeable higher overlap between clusters when all mapped piRNAs were considered (Figure S7A). Indeed, the three major germline clusters were expected to provide for most piRNAs (>50%) for 17 TE families (Table S4). Therefore, despite the known redundancy due to the repetitive nature of TEs and their distribution across the genome, our analyses indicate the existence of a structured and non-redundant relationship between large germline piRNA clusters and TE families.

To directly test the predicted cluster-TE relationships, we used the small RNA data generated from ovaries of germline piRNA cluster mutants. First, the analyses revealed that cluster deletions affected piRNA production in *cis*, with little compensatory or *trans* effect observed in mutants compared with the *w*^*1118*^ control (Figure 5B; Figures S7B–S7D). Second, loss of uniquely mapping piRNAs largely mirrored the predicted cluster-TE relationship, with triple mutants lacking the majority of unique piRNAs (>75%) for 24 of the 28 expected families (Figure 5C). A similar but less pronounced correlation was observed when all mapped piRNAs were considered (Figure S7E). Moreover, only minor changes were observed in the accumulation of piRNAs originated from dispersed copies found outside the deleted clusters (Figure S7C). The few exceptions involved a small fraction of TE families targeted by the deleted piRNA clusters, likely revealing varying degrees of feedback between clusters and dispersed copies. Most important, however, complete to nearly complete loss of all piRNAs (>75%) was observed for 23 TE families in triple mutants compared with *w*^*1118*^ control ovaries (Figure 5D; Figures S7D–S7E). This was associated with a general strong loss of total piRNAs targeting TEs in gonads, which was progressively reduced from single to double mutants and reached a total of 42% loss in triple mutants (Figure 5E; Figure S7F). In comparison, germline-specific knockdown of *piwi* led to a reduction of ∼15% in the total amount of TE-matching piRNAs, while the knockdown of ping-pong cycle proteins *aub* and *ago3* was associated with a loss of 55%–60% of piRNAs (Figure 5E; Senti et al., 2015). Altogether, these results confirmed the existence of a non-redundant relationship between large germline piRNA clusters and TE families. Surprisingly, despite the lack of developmental phenotypic changes in mutants, our results revealed that large germline piRNA clusters directly contribute to a large percentage of TE-derived piRNAs.

### Loss of major germline piRNA clusters does not entail transposon reactivation, increased transposition, or changes in gene expression

**Figure 6:**
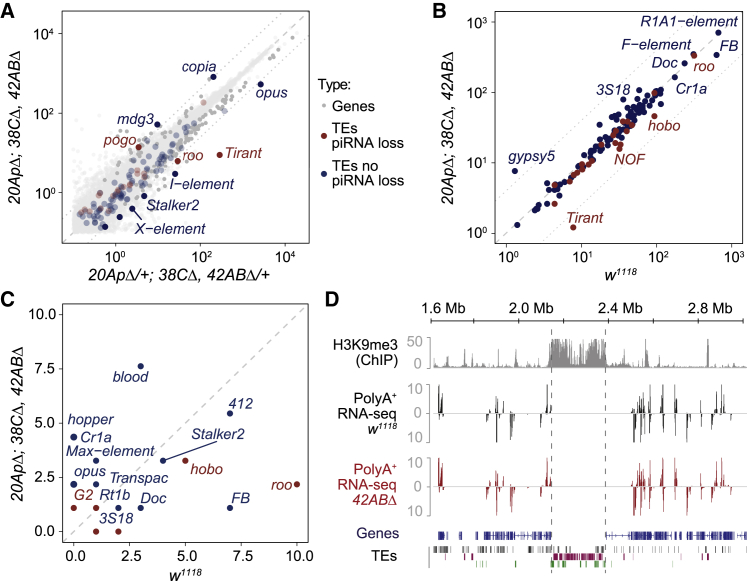
Impact of piRNA cluster deletions on the activity of TEs and neighboring genes (A) Scatterplot showing the expression of genes (gray dots), transposons with >75% piRNA loss in triple mutants (red dots), and all other transposon families (blue dots) as measured by RNA-seq analysis (expressed in fragments per kilobase per million fragments [FPKM], log_10_), in *20A(p)Δ/+; 38CΔ/+, 42ABΔ/+* heterozygous versus *20A(p)Δ; 38CΔ, 42ABΔ* homozygous mutant ovaries. Dashed line represents perfect x = y correlation. Dotted lines indicate 4-fold change. (B) Scatterplot showing the genomic copy number for each TE family in triple-mutant flies (*20A(p)Δ; 38CΔ, 42ABΔ*) compared with *w1118* control as measured by DNA-seq data analysis (expressed in read base pairs per TE base pairs divided by genomic coverage depth, log_10_). (C) Scatterplot showing the number of non-reference TE insertions in triple-mutant flies (*20A(p)Δ; 38CΔ, 42ABΔ*) compared with *w1118* control as measured by DNA-seq data analysis. (D) Density plots for normalized strand-specific mRNA steady-state levels (measured by RNA-seq and represented as reads per million [RPM]) in the vicinity of the *42AB* piRNA cluster locus in *42ABΔ* mutant ovaries compared with *w1118* control. The top track shows H3K9me3 ChIP-seq signal in control ovaries. Annotation is at the bottom: genes (blue), DNA transposons (black), LTR retrotransposons (purple), and non-LTR retrotransposons (green). See also Figures S8 and S9.

To determine the effect of cluster deletions on gene and TE expression, we performed RNA-seq analyses with poly-A selected mRNAs extracted from adult ovaries. Surprisingly, our results indicated that the accumulation of TE mRNAs was mostly unchanged in homozygous mutants compared with the respective heterozygotes (Figure 6A), even when TE families that have lost >75% of all piRNAs in triple mutants were considered. Likewise, no significant changes were observed in RNA-seq comparisons between double-homozygous mutants and heterozygotes (Figure S8A). Exceptions were observed only when mutants were directly compared with *w*^*1118*^, but considering the direction of change and the TE families involved, these are likely to reflect background differences (Figure S8B). These results contrast with the strong upregulation of transposon transcripts in the ovaries of germline-specific knockdown for piRNA biogenesis proteins such as *piwi*, *aub*, and *Argonaute3* (Rozhkov et al., 2013; Senti et al., 2015). Therefore, and against the prediction of piRNA cluster-mediated TE control, we conclude that the loss of the three major germline piRNA clusters does not lead to an increase in the accumulation of transposon transcripts.

To investigate whether the loss of germline piRNA clusters led to a burst in transposon activity or an increase in transposon copy number, we performed DNA-seq analysis and cataloged the transposon insertions in *w*^*1118*^ female controls and triple-mutant females obtained from a stock kept in homozygosity for more than 2 years. Our bioinformatic analysis revealed small differences in total TE copy number between triple mutants and *w*^*1118*^ controls (Figure 6B), and the few exceptions involved families that were not related to the germline piRNA clusters studied here (such as *Tirant* and *Gypsy5*) and therefore likely reflect pre-existing background differences. Importantly, transposon families that have lost >75% of piRNAs in triple mutants did not show any significant increase in copy number. To further explore this, we focused on the TE insertions that are not present in the original *D. melanogaster* genome assembly (Figure 6C), as these are likely to represent new transposition events. Our results revealed the existence of a few changes that were randomly distributed in the two backgrounds. Similar to what was observed for the total TE copy number, we observed no correlation between families that lose piRNAs in triple mutants and new transposition events. Collectively, our results indicate that disruption of the major germline piRNA clusters and consequent piRNA loss does not entail a change in transposon activity in *trans*.

Major germline piRNA clusters consist of large blocks of heterochromatin surrounded by genes (Figure 6D; Andersen et al., 2017; Brennecke et al., 2007; Klattenhoff et al., 2009). Given the effect of heterochromatic marks on nearby gene expression and the well-documented position-effect variegation (PEV) effect when genes are found juxtaposed with heterochromatin (Elgin and Reuter, 2013), we analyzed the transcriptome of mutant and control flies for changes in gene expression. The analysis indicated that gene expression in general, as well as at regions flanking the *38C* and *42AB* deletions, was similar in mutants and *w*^*1118*^ controls (Figures 6A and 6D), with the few genes showing statistically significant changes being randomly distributed across the genome (data not shown). Therefore, these results revealed that the heterochromatic state of the large piRNA clusters does not interfere with the expression of either adjacent or distal genes. Similarly, we also concluded that the transposon sequences found within *42AB* and *38C* are unlikely to provide for enhancers or other regulatory sequences that could control nearby gene expression.

### Analysis of germline piRNA clusters and PIWI-regulated transposon families

Despite the drastic loss of piRNAs for 23 TE families in the triple mutants (Figure 5D), we observed no correlational change in TE transcript accumulation in mutants compared with controls (Figure 6A). In contrast, germline-specific knockdown of the PIWI proteins has been shown to lead to a robust increase in transcriptional activity and transcript accumulation for a total of 24 TE families (excluding the three telomeric-associated TE families *Het-A*, *TAHRE*, and *TART* (Senti et al., 2015). With the notable exception of *gypsy12*, for which the only mostly intact full-length copy present in the genome is found at the *42AB* locus, the comparison between the two groups revealed no overlap (Figures S9A and S9C). PIWI-regulated TEs are enriched for LTR retrotransposons (Senti et al., 2015), while the 23 TE families with a drastic loss of piRNAs in triple mutants showed an even distribution of DNA transposon and retrotransposon families (LTR and non-LTR). With one exception (*GATE*), PIWI-regulated TEs were consistently present in multiple full-length copies in the *D. melanogaster* genome (Kaminker et al., 2002), and the accumulation of piRNAs for such families was mostly unchanged in triple mutants (Figures S7D and S9B). On the other hand, 7 of the 23 families losing >75% of piRNAs in triple mutants lack full-length insertions in the genome (*G4*, *Helena*, *NOF*, *rooA*, *transib1*, *transib2*, *transib3*) and are present only as fragmented copies. Although the absence of full-length copies could explain the lack of upregulation upon loss of piRNAs for these families, 16 other TE families that rely on the major germline clusters for piRNA production were consistently present in full-length copies in the genome (Figure S9B; Hoskins et al., 2015; Kaminker et al., 2002).

**Figure 7:**
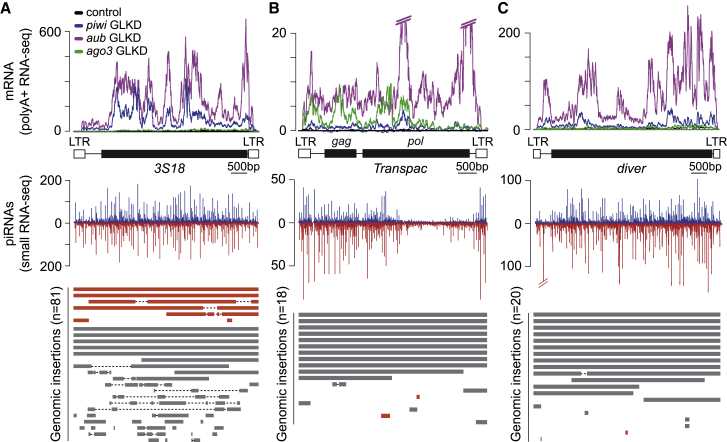
Examples of TEs with cluster-independent piRNA production (A–C) Top graphs: density plots for normalized strand-specific mRNA steady-state levels (measured by RNA-seq and represented as reads per million [RPM]) over TE consensus in control (black) and germline knockdown mutants for *piwi* (blue), *aub* (purple), and *ago3* (green; Senti et al., 2015). Middle graphs: density plots for normalized piRNA read (measured by small RNA-seq and represented as reads per million [RPM]) over TE consensus in *w1118* ovaries. Bottom panels: distribution of genomic insertions aligned over TE consensus sequence. Insertions found within piRNA clusters are shown in orange. Insertions dispersed outside of piRNA clusters are represented in gray. (A) *3S18*, (B) *Transpac*, and (C) *Diver*.

Given that the major germline piRNA clusters were dispensable for PIWI-mediated TE silencing, we investigated the genomic distribution for the 24 TE families shown to be regulated by PIWI proteins in the germline (excluding telomeric-associated TE families). For 20 of 24 families, such as *3S18*, the genomic distribution revealed that fragmented and full-length copies were found both within or outside piRNA clusters (considering all piRNA clusters; Brennecke et al., 2007) and in many cases were present in multiple piRNA clusters (Figure 7A). However, for other TE families shown to be regulated by PIWI proteins such as *Transpac*, *diver*, *flea*, and *jockey*, we observed that the majority of genomic copies were found dispersed in the genome and were rarely found inside piRNA clusters (Figures 7B and 7C). In such cases, TE insertions found within piRNA clusters were consistently small and fragmented. Given this and the observed distribution of piRNAs over full-length elements (Figure 7), our analyses indicate that the primary source for piRNA production for such families is provided by dispersed full-length TE insertions rather than the germline piRNA clusters.

## Discussion

Using genomic analysis, we identify hundreds of piRNA clusters in the *Drosophila* genus and found that, similar to *D. melanogaster*, a few large, TE-dense loci produce the majority of unique piRNAs in the analyzed species. However, our evolutionary analysis revealed that, with a few exceptions such as *42AB* and *38C*, most *Drosophila* germline piRNA clusters are species specific, with an estimated age ranging between 0 and 4 My. In agreement, TEs within piRNA clusters are relatively younger compared with both heterochromatic and euchromatic compartments. Hence, *Drosophila* germline piRNA clusters seem evolutionary labile, especially compared with pachytene piRNA clusters in mammals. Indeed, most distantly related primate species, which average 65–75 My distance, share about a third of their pachytene piRNA clusters (Gebert et al., 2019). Eutherian mammals, a phylogenetic group that is approximately 150 My old, share a core of about 20 pachytene piRNA-producing loci (Chirn et al., 2015). Interestingly, promoter deletions of evolutionarily conserved pachytene piRNA clusters resulted in mixed outcomes in terms of fertility, with deletion of the *pi6* locus in mice leading to sterility, while mutants affecting the *p17* locus show no phenotype (Wu et al., 2020).

*Drosophila* piRNA clusters are different from mammalian pachytene counterparts in many aspects, such as TE content, mode of transcription, and genomic distribution (Andersen et al., 2017; Brennecke et al., 2007; Özata et al., 2020; Wu et al., 2020). Particularly, the high TE density and heterochromatic nature of *Drosophila* piRNA clusters, as opposed to TE depletion and euchromatic localization of mammalian pachytene piRNA clusters, suggest fundamental differences in emergence, maintenance, and turnover. In this context, it might be expected that TE-dense heterochromatic loci with non-canonical modes of transcription, such as observed in the *Drosophila* germline, would be predicted to be far less conserved than euchromatic piRNA loci with canonical transcription found in mammals. Moreover, mutation rates have been shown to be dependent on the number of generations or germ cell divisions rather than on absolute time, meaning that the number of mutations in a given time frame is about an order of magnitude higher in flies than in mice (Drost and Lee, 1995).

Surprisingly, deletion of the three most highly expressed germline piRNA loci in *D. melanogaster* (*42AB*, *38C*, and *20A*) affected neither fertility nor TE mobilization despite a considerable loss (>40%) of all TE-matching piRNAs. Even the most affected TE families, which lost almost all cognate piRNAs, did not show any signs of de-repression. This indicates that either silencing of many TE families that are primary targets of *42AB*, *38C,* and *20A* does not depend on the germline piRNA pathway or that such TE families have no transcriptionally competent copies in the genome of the studied strain. On the one hand, this implies that a large fraction of germline-accumulating piRNAs may be superfluous and irrelevant for TE control (Kelleher and Barbash, 2013). On the other hand, our results also suggest that a number of these TE families that contain full-length copies outside the deleted clusters are either not transcriptionally competent, despite the accumulation of cognate piRNAs, or that their silencing is imposed by PIWI-independent mechanisms (W. Theurkauf, personal communication).

The piRNA cluster *flamenco*, which is specifically expressed in somatic follicle cells, is required for female fertility and transposon control (Brennecke et al., 2007; Pélisson et al., 1994). However, our findings indicate that the model by which piRNA clusters act as principal regulators of transposon activity in *trans* is at least partly inaccurate in the context of the germline. The “jumping-in-cluster” model, in which germline piRNA clusters gain the ability to target active TEs in *trans* upon random integration, is not supported by recent findings that indicate that TEs do not show a tendency for inserting into piRNA clusters (Moon et al., 2018). Although studies surveilling transposon invasion suggested that trapping into germline piRNA clusters would be required for piRNA production and silencing in the germline (Duc et al., 2019; Khurana et al., 2011), our study suggests an alternative model that the more numerous insertions dispersed across the genome may contribute to piRNA production. Indeed, a recent study suggested that *de novo* induction of piRNA biogenesis at dispersed insertion sites is more prevalent than piRNA cluster trapping in natural populations of *D. melanogaster* (Luo et al., 2020). As germline piRNA clusters do not seem to regulate TE in *trans*, young active TE insertions such as the *gypsy12* copy found at *42AB* may be the actual entities that are regulated by the piRNA pathway in *cis*. It has been previously proposed that transcriptionally active TE insertions can be converted into Rhino-dependent piRNA-producing loci upon targeting by Piwi-bound piRNAs (Andersen et al., 2017; Mohn et al., 2014), irrespective of their location. In support of this, we identified PIWI-regulated TE families for which the bulk of piRNAs are provided by dispersed, stand-alone full-length insertions rather than piRNA clusters. This is reminiscent of what is observed in mammals, in which dispersed transposon copies are responsible for producing primary piRNAs (Aravin et al., 2008). In this context, a yet unknown mechanism, rather than insertions into an existing cluster, would be responsible for initiating the targeting of newly invading TEs by the piRNA machinery. In plants, it has been suggested that new TEs are sensed by the plant transposon-targeting small RNA system after their copy number and expression exceeds a certain threshold (Hirochika et al., 2000; Marí-Ordóñez et al., 2013; Pérez-Hormaeche et al., 2008). In this case, however, it is unclear how such a mechanism would distinguish newly invading TEs from highly expressed genes. Although mounting evidence points to a role for TE copies dispersed throughout the genome in mediating silencing in *cis*, it is nonetheless possible that small piRNA clusters, rather than the large clusters studied here, may collectively contribute to silencing of TEs in *trans*.

We identified *42AB* and *38C* as the most conserved piRNA clusters among the ten *Drosophila* species analyzed in this study. This is surprising given our evidence that these clusters are dispensable for TE regulation. Although conservation is usually associated with functional relevance, it is possible that these two loci may be beyond their evolutionary time window of critical function and that their demise could be imminent, as the typical lifespan of *Drosophila* germline piRNA clusters appears to be very short. On the other hand, the uni-strand *20A* cluster likely emerged very recently, which is supported by its absence from closely related species and by its exceptionally low TE sequence divergence (∼3%). Moreover, *20A* has a stereotypical structure, with virtually all TEs oriented in the antisense direction to the accumulation of piRNAs. Despite that, disruption of *20A* transcription by promoter deletion did not affect TE repression in *cis* or in *trans*. Although the evolution of structured uni-strand clusters is likely to be determined by mechanistic constraints and/or strong selective pressure that could be manifested in a *cis*- and/or *trans*-regulatory capacity, our results suggest that the window of evolutionary benefit for clusters may be very narrow and has potentially already passed for the *20A* locus.

Given that large germline piRNA clusters seem to have no effect in *trans* and may only regulate TE expression in *cis*, their recurrent emergence and structural expansion in *Drosophila* genomes, followed by a seemingly fast demise, poses another interesting and unsolved puzzle. The fact that piRNA clusters arise at genomic loci marked by recurrent inversion breakpoints, as well as that they are commonly located in close proximity to pericentromeric heterochromatin, might provide some clues. It is known that chromosomal inversions can result in a reduction in recombination frequency at the breakpoints and that this effect is most prominent in the proximity to the centromere (Corbett-Detig and Hartl, 2012; Farré et al., 2013). In general, pericentromeric regions show low recombination rates (Hughes et al., 2018) and TEs are known to accumulate in regions of low recombination (Dolgin and Charlesworth, 2008; Kent et al., 2017; Rizzon et al., 2002). Therefore, the proximity to pericentromeric regions might favor the buildup of TE clusters that are not immediately purged from the genome through recombination but preserved to generate *cis*-regulating piRNA-producing loci. Their subsequent relatively quick loss on an evolutionary timescale might then be of no consequence, a conclusion supported by the lack of molecular or organismal phenotypes observed when the major germline piRNA clusters are deleted in *D. melanogaster*.

### Limitations of the study

All the experiments described in this study were performed with lab strains, and piRNA cluster deletions were generated by combining multiple lines with different backgrounds. However, it is worth noting that given the rather dynamic nature of TEs in natural populations and over short evolutionary time (Petrov et al., 2011), it is nonetheless possible that the studied clusters may still be functionally relevant for fertility and TE regulation in other *D. melanogaster* strains or in wild populations. Furthermore, small RNA-seq analyses were normalized by the number of miRNA-matching reads, on the basis of the assumption that miRNA pools are constant in the analyzed samples. Finally, all small RNA-seq analyses were performed on n = 1.

## STAR★Methods

### Key resources table

**Table:** 

REAGENT or RESOURCE	SOURCE	IDENTIFIER
**Chemicals, peptides, and recombinant proteins**		

TRIzol™ reagent	ThermoFisher Scientific	Cat# 15596026
RQ1 RNase-Free DNase	Promega	Cat# M6101
Vectashield® media containing DAPI	Vector Laboratories	Cat# H-1200

**Critical commercial assays**		

Quick-DNA Microprep Kit w/ Zymo-Spin	Zymo Research	Cat# D3020
Nextera DNA Flex Library Prep kit	Illumina	Cat# 20018704
NEBNext® Poly(A) mRNA Magnetic Isolation Module	NEB	Cat# E7490
NEBNext® Ultra™ Directional RNA Library Prep Kit for Illumina®	NEB	Cat# E7420
NEBNext® Multiplex Oligos for Illumina®	NEB	Cat# E7500/E7600
NEBNext® Small RNA Library Prep Set for Illumina®	NEB	Cat# E7330
Qubit dsDNA HS Assay Kit	ThermoFisher Scientific	Cat# Q32851
Qubit RNA HS Assay Kit	ThermoFisher Scientific	Cat# Q32852

**Deposited data**		

High-throughput Sequencing (DNA-seq, RNA-seq, small RNA-seq)	This study; GEO	GEO: GSE174561
Confocal images	This study; Mendeley Data	Mendeley Data: https://data.mendeley.com/datasets/8vkjt29b4f/1
Nanopore-sequenced genome assemblies	(Miller et al., 2018)	https://github.com/danrdanny/Drosophila15GenomesProject/
Nanopore-sequenced genome assemblies	(Shah et al., 2019)	NCBI Bioproject: PRJNA515844
Small RNA-seq data	(Barckmann et al., 2015)	GEO: GSM1818089
Small RNA-seq data	(Mohammed et al., 2018)	GEO: GSE98013
Small RNA-seq and RNA-seq data	(Senti et al., 2015)	GEO: GSE71775
Small RNA-seq data	(Olivieri et al., 2012)	GEO: GSE38728

**Experimental models: Organisms/strains**		

*D. melanogaster: w*^*1118*^	Ruth Lehmann lab	N/A
*D. melanogaster: w*^*1118*^*P{XP}d03497*	Harvard Exelixis Stock Collection	Exelixis# d03497
*D. melanogaster: w1118 PBac{WH}f02310*	Harvard Exelixis Stock Collection	Exelixis# f02310
*D. melanogaster: Df(1)BSC588 w*^*1118*^*/Binsinscy*	Bloomington Drosophila Stock Center	BDSC# 25422
*D. melanogaster: Df(1)Exel6255 w*^*1118*^*/FM7c*	Bloomington Drosophila Stock Center	BDSC# 7723
*D. melanogaster: Dp(1;Y)BSC344 y*^*+*^*P{w*^*+*^*}BSC28 B*^*S*^*/winscy/C(1)RA In(1)sc*^*J1*^*In(1)sc*^*8*^*l(1)1Ac*^*1*^	Bloomington Drosophila Stock Center	BDSC# 36485
*D. melanogaster: w*^*1118*^*; P{RS3}CB-6748-3*	Kyoto Stock Center (DGRC)	Kyoto# 124205
*D. melanogaster: w*^*1118*^*; P{RS5}5-SZ-4007*	Kyoto Stock Center (DGRC)	Kyoto# 126282
*D. melanogaster: w*^*1118*^*; PBac{RB}e04172*	Harvard Exelixis Stock Collection	Exelixis# e04172
*D. melanogaster: w*^*1118*^*; P{XP}d00877*	Harvard Exelixis Stock Collection	Exelixis# d00877
*D. melanogaster: w*^*1118*^*;; MKRS, P{hsFLP}86E/TM6B, Tb*^*1*^	Bloomington Drosophila Stock Center	BDSC# 279
*D. melanogaster: FM7h/Dp(2;Y)G, P{hs-hid}Y*	Bloomington Drosophila Stock Center	BDSC# 23661
*D. melanogaster: y w P{ry[+] FLP22}; If/CyO, hs-hid*	Ruth Lehmann lab	N/A
*D. melanogaster: w*^*1118*^*; aub*^*QC42*^*cn*^*1*^*bw*^*1*^*/CyO P{sevRas1.V12}FK*	Bloomington Drosophila Stock Center	BDSC# 4968
*D. melanogaster: aub*^*HN2*^*cn*^*1*^*bw*^*1*^*/CyO*	Paul Macdonald lab	N/A
*D. melanogaster: P{ry[+t7.2] = PZ}rhi[02086] cn*^*1*^*/CyO; ry*^*506*^	Bloomington Drosophila Stock Center	BDSC# 12226
*D. melanogaster: w*^*1118*^*; Df(2R)Exel7149/CyO*	Bloomington Drosophila Stock Center	BDSC# 7890

**Software and algorithms**		

Unitas	(Gebert et al., 2017)	https://sourceforge.net/projects/unitas/
mirDeep2	(Friedländer et al., 2012)	https://github.com/rajewsky-lab/mirdeep2
RepeatModeler	N/A	https://www.repeatmasker.org/RepeatModeler/
RepeatMasker	N/A	https://www.repeatmasker.org/
proTRAC	(Rosenkranz and Zischler, 2012)	https://sourceforge.net/projects/protrac/
Integrative Genomics Viewer IGV	(Robinson et al., 2011)	https://igv.org/
piPipes package	(Han et al., 2015)	https://github.com/bowhan/piPipes
Custom Perl and R scripts	This study	https://zenodo.org/record/5085862

**Other**		

Custom Stellaris® RNA FISH Probes (Quasar670)	This study - Table S5	N/A

### Resource availability

#### Lead contact

Further information and requests for resources and reagents should be directed to and will be fulfilled by the lead contact, Felipe Karam Teixeira (fk319@cam.ac.uk).

#### Materials availability

*Drosophila* stocks generated in this study are available from the lead contact without restrictions.

### Experimental model and subject details

#### *Drosophila* genetics and husbandry

All stocks and crosses were maintained at 25°C on standard medium, and fly strains used in this study are listed in the key resources table. Genetic crosses to combine the chromosomal deletions overlapping over the *20A* were performed with the deletion stocks *Df(1)BSC588 w*^*1118*^*/Binsinscy* and *Df(1)Exel6255 w*^*1118*^*/FM7c*, as well as the duplication stock *Dp(1;Y)BSC344 y*^*+*^ *P{w*^*+*^*}BSC28 B*^*S*^*/winscy/C(1)RA In(1)sc*^*J1*^ *In(1)sc*^*8*^ *l(1)1Ac*^*1*^. Chromosome deletions were generated as previously described (Ryder et al., 2004; Thibault et al., 2004). Briefly, for the *20A* promoter deletion (chrX:21390230-21391839, dm3), FRT-bearing *P*-element insertions from stocks *w*^*1118*^ *P{XP}d03497* and *w*^*1118*^ *PBac{WH}f02310* were recombined using *w*^*1118*^*;; MKRS, P{hsFLP}86E/TM6B, Tb*^*1*^ as a FLP source, and the resulting deletion was balanced using the *FM7h/Dp(2;Y)G, P{hs-hid}Y* stock. For the deletion encompassing the *38C* locus (chr2L:20104769-20243057, dm3), FRT-bearing *P*-element insertions from the stocks *w*^*1118*^*; P{RS3}CB-6748-3* and *w*^*1118*^*; P{RS5}5-SZ-4007* were recombined using *y w P{ry[+] FLP22}; If/CyO, hs-hid* as a FLP source. The same process was performed to recombined the FRT-bearing *P*-element insertions from the stocks *w*^*1118*^*; PBac{RB}e04172* and *w*^*1118*^*; P{XP}d00877*, which resulted in the *42AB* locus deletion (chr2R:2159264-2389366, dm3). Double and triple mutants were generated by recombination.

Experiments were performed with 3- to 5-day-old adult female flies. For the fertility tests displayed in Figure 4, homozygous female virgins obtained from homozygous mothers were mated to *w*^*1118*^ males, and eggs were collected in agar plates. For egg-laying experiments, parents were flipped to a new agar plate every ∼12 hours, and the number of eggs/day was determined as the average of eggs laid for 24 hours over 3 consecutive days. For egg hatching experiments, agar plates with eggs were kept at 25°C for another ∼28 hours prior to counting. All experiments were performed in at least two biological replicates.

### Method details

#### Analysis of piRNA cluster evolution

##### Small RNA-seq data processing

For the analysis of piRNA cluster evolution in the *Drosophila* genus, we took advantage of published small RNA-seq datasets (Barckmann et al., 2015; Mohammed et al., 2018) generated from whole embryos of 10 different *Drosophila* species (*D. melanogaster*, *D. sechellia*, *D. simulans*, *D. yakuba*, *D. ananassae*, *D. pseudoobscura*, *D. persimilis*, *D. willistoni*, *D. mojavensis*, *D. virilis*). After quality control with FastQC (Andrews, 2010), non-coding RNA and cDNA sequences were filtered with unitas (Gebert et al., 2017), using the total set of ncRNAs (FlyBase) as well as rRNA sequences (NCBI nucleotide database) from the respective *Drosophila* species. In parallel, novel miRNA sequences were predicted with mirDeep2 (Friedländer et al., 2012) and filtered from the resulting matches. After sequence filtering, small RNAs with a length between 23 and 29nt were mapped to the corresponding genome.

#### Genome mapping

Nanopore-sequenced genome contigs < 50kb were removed prior to mapping (Miller et al., 2018; Shah et al., 2019). Filtered small RNA reads were first mapped with bowtie (Langmead et al., 2009), allowing no mismatches and discarding multi-mappers. Second, small RNA reads were re-mapped to allow all valid best alignments, i.e., including multi-mappers. The resulting alignments were scanned for highly structured loci, which were removed from the map files. Both alignment files were analyzed with unitas (Gebert et al., 2017), producing information on read length distribution, nucleotide frequencies, and ping-pong signatures.

#### Repeat annotation

*De-novo* transposon identification and annotation was performed for the nanopore-sequenced genome assemblies for all 10 *Drosophila* species used in this study. First, the software package RepeatModeler was employed for the identification of transposable elements, including previously unknown families. The resulting outputs were then used to scan for transposable element insertions with RepeatMasker. All subsequent analyses of repeat contents were conducted with custom Perl and R scripts (scripts and annotations available at https://zenodo.org/record/5085862).

#### Identification of piRNA clusters

Identification of germline piRNA clusters was achieved using uniquely mapping piRNA-like small RNAs and the software tool proTRAC (Rosenkranz and Zischler, 2012). A minimum cluster size of 5 kb and a minimum rate of 1U or 10A of 30% (while ignoring strand distribution) was used for cluster identification, as well as a *p-value* of 0.05 for read density. Closely adjacent clusters were merged if the gap between two loci was smaller than their combined length. For each identified piRNA cluster, ping-pong z-scores for uniquely-mapping reads and all reads were calculated. Finally, additional information such as TE content and strandedness of reads and TE insertions was integrated using the custom RepeatMasker annotations described here (https://zenodo.org/record/5085862).

#### Analysis of transposon sequence divergence

Approximation of the Kimura divergence (Kimura, 1980) was used to calculate sequence divergence from transposon consensus sequence for each repeat insertion found at piRNA clusters and in the rest of the genome. Average transposon divergence at cluster border regions was determined using 0.5 kb windows. Average for each window over all regions was then determined after obtaining the mean divergence for each window of each cluster. For the calculation of mean transposon sequence divergence on different genomic compartments, heterochromatin and euchromatin were divided by the density of transposon insertions. After manual validation, contiguous regions with two-fold transposon density above genomic average reaching at least one chromosome/contig end were designated as heterochromatin.

#### Evolutionary analysis of piRNA clusters

The synteny-based, pairwise species search for homology in non-annotated, nanopore-sequenced genomes (Miller et al., 2018; Shah et al., 2019) was performed by focusing on the 200kb upstream and downstream of the piRNA cluster border coordinates. These were then used for blastn searches (Camacho et al., 2009) to find the respective regions in annotated, but less contiguous, genome assemblies (NCBI Genome; Drosophila 12 Genomes Consortium et al., 2007). The corresponding gene sequences, as obtained from GFF files, were then searched in nanopore-sequenced genome assemblies with blastn to determine their exact coordinates.

Using the information on *Drosophila* gene orthologs obtained from FlyBase, sequences of orthologs were extracted in a pairwise comparison and used for blastn searches to find the corresponding location in their nanopore-sequenced genome assembly. Loci for which both homologous flanks were located on the same chromosome/contig were favored as most likely syntenic region, as well as those that are closest toward the piRNA cluster of the query species, and finally the longest contiguous flanks. In Figure 2E, a given data point was considered as an “inversion break” if the synteny in the analyzed species could be followed but was found to be broken at the specific location (between the two flanking genes) where the piRNA cluster was identified in the original species. In cases of unbroken synteny, i.e., if the supposed homologous location of a piRNA cluster can be conclusively inferred by the gene sequence and orientation of the flanking genes (“active piC” or “synteny”), data on transposon content and piRNA expression measured in reads per million was included to check for the presence of an active piRNA cluster. In this case, thresholds were of 100 rpm and 10 rpkm for piRNA expression, minimum size of 5 kb, as well as minimum transposon content of 25% and maximum gene content of 25% (https://zenodo.org/record/5085862). All cases have been manually reviewed to minimize false positive and false negative decisions.

Homologies in each possible pair of species among the ten species were then analyzed, resulting in a total of 90 pairwise comparisons. On that basis, homologies across all ten species were determined through the linking of overlapping loci between pairwise comparisons.

#### Rearrangement analysis

Identification of chromosomal rearrangements at syntenic regions was performed as described in Alekseyev and Pevzner, 2007, in which the flanking gene arrays of the subject species were represented according to the order and direction (+ or -) of the homologous genes in the query species as signed permutations. Breakpoints were identified by adjacencies, namely the distance of two consecutive elements in a signed permutation (p_i+1_ - p_i_), that are unequal to 1. Inversion breakpoints were then determined by sign reversals at disordered adjacencies as previously described (Alekseyev and Pevzner, 2007). To determine the background frequency of inversion breakpoints expected in the analyzed genomes, we generated 10 random sets of genomic loci, each corresponding to the number of evolutionarily traced loci in the set of piRNA clusters for the 10 studied *Drosophila* species. For statistical analysis, z-scores were calculated for the number of breakpoints between flanks compared to the background distribution in the flanking regions. Z-scores (Z0) were calculated for the breakpoint frequency between flanks (x0) using the formula Z0 = (x0-μ)/σ, with flank breakpoint frequency mean μ and standard deviation σ.

#### DNA-sequencing analyses

Genomic DNA was obtained from 20-40 female adult flies from *w*^*1118*^ and a triple mutant (*20A(p)Δ*; *38CΔ, 42ABΔ*) stock kept in homozygosity for over 2 years. DNA was extracted using the Quick-DNA Microprep Kit w/ Zymo-Spin and quantified using Qubit dsDNA HS Assay Kit. Library preparation was performed on 0.25μg of genomic DNA using the Nextera DNA Flex Library Prep kit as described by the manufacturer (Illumina). Libraries were multiplexed and sequenced in paired-end, 150-nt-long reads on an Illumina NovaSeq.

Paired-end reads were mapped to the *Drosophila melanogaster* genome (dm3) using BWA MEM (Li and Durbin, 2009), allowing only primary alignments. The resulting SAM/BAM files were then filtered to retain only uniquely mapping reads using Samtools (Li et al., 2009b) and the command line utility grep, including reads without alternative hits or other alignments (tags XA:Z and SA:Z). Additionally, a map quality filter with a threshold value of 60 was applied with Samtools. Subsequently, bigwig files were created with Bedtools (Quinlan and Hall, 2010) and the UCSC genome browser command-line tool bedGraphToBigWig for coverage visualization with the Integrative Genomics Viewer IGV (Robinson et al., 2011). The (k,e) mappability of the *D. melanogaster* genome assembly dm3 was computed with genmap (Pockrandt et al., 2020), using a standard ‘k’ of 30 and an ‘e’ of 2.

The analysis of total genomic TE copy numbers was conducted by mapping paired-end reads to the complete set of *D. melanogaster* TE consensus sequences (FlyBase) using bowtie2 and limiting the number of distinct valid alignments to one per read (‘-k 1’). Read counts for each TE family were normalized by genomic coverage as follows: read counts were multiplied by 150 nt paired-end read base pairs (300), divided by TE consensus sequence length and genomic read coverage. In parallel, non-reference TE insertions were identified with TEMP (Zhuang et al., 2014) using DNA-seq read alignments on *D. melanogaster* genome (dm3) generated by the BWA ALN algorithm. To avoid false positives, TEMP output was filtered to discard insertions with ‘population frequencies’ lower than 10.

#### RNA-sequencing analyses

Total RNA from dissected adult ovaries was isolated using TRIzol™ reagent and quantified using Qubit RNA HS Assay Kit. Contaminating DNA was removed using RQ1 RNase-Free DNase as described by the manufacturer (Promega). Poly(A)-selected RNA-sequencing (RNA-seq) analysis was performed on 2.5μg of total RNA using the NEBNext® Poly(A) mRNA Magnetic Isolation Module and the NEBNext® UltraTM Directional RNA Library Prep Kit for Illumina®. Libraries were multiplexed using the NEBNext® Multiplex Oligos for Illumina® and sequenced in single-end, 50-nt-long reads on an Illumina HiSeq 2500.

RNA-seq data were mapped to the *Drosophila melanogaster* genome (dm3) and the FlyBase and Repbase transposon consensus database using the piPipes package (version 1.5.0; https://github.com/bowhan/piPipes), following the RNA-seq pipeline (Han et al., 2015). Briefly, libraries were aligned to ribosomal RNA using Bowtie2 (Langmead and Salzberg, 2012), and non-rRNA-mapping reads were then mapped to the transcriptome and transposon consensus using Bowtie2. Transposon transcript abundance was quantified using eXpress (Roberts and Pachter, 2013) and differentially gene expression analysis was performed using Cuffdiff (Trapnell et al., 2013), following the default settings on the piPipes package (Han et al., 2015). Analyses were performed with two samples, each with two biological replicates, using the piPipes RNA-seq dual-library mode (Han et al., 2015). For the analysis of germline knock-downs for *piwi*, *aub,* and *ago3*, raw RNA-seq datasets were retrieved from the GEO database, accession number GSE71775 (Senti et al., 2015).

#### Small RNA-sequencing analyses

Total RNA from dissected adult ovaries was isolated using Trizol reagent (Invitrogen) and quantified using Qubit (Invitrogen). Briefly, small RNA-sequencing analysis was performed on 10 μg of total RNA using the NEBNext® Small RNA Library Prep Set for Illumina® and with an initial 2S rRNA depletion step as previously described (Li et al., 2009a). Libraries were multiplexed using the NEBNext® Multiplex Oligos for Illumina® and sequenced in single-end, 50-nt-long reads on an Illumina HiSeq 2500.

Small RNA sequencing reads were quality-filtered and trimmed with Trim-Galore, applying standard settings. For cross-sample normalization, trimmed reads were mapped to the complete set of mature miRNA sequences of *D. melanogaster* (miRBase; Kozomara and Griffiths-Jones, 2011) with bowtie (Langmead et al., 2009), reporting all best valid alignments without mismatches. In parallel, trimmed reads were mapped to the combined set of non-coding RNA, protein-coding, and pseudogene sequences (FlyBase) allowing two mismatches and retaining unmatched reads in the size range of 23-29 nt. Filtered reads were then mapped to the *D. melanogaster* genome (dm3) using bowtie and allowing either perfect unique matches or all best valid alignments without mismatches.

For the analysis of piRNA cluster expression, we determined the number of genome-mapped reads located in clusters that were initially identified by Brennecke and colleagues (Brennecke et al., 2007). Similarly, we calculated numbers of TE-matching genome-mapped reads using the corresponding RepeatMasker output. To allow for comparison between samples, all read counts were normalized by the previously discerned number of miRNA-matching reads. For the analysis of germline knock-downs for *piwi*, *aub,* and *ago3*, raw small RNA-seq datasets were retrieved from the GEO database, accession numbers GEO: GSE71775 (Senti et al., 2015) and GEO: GSE38728 (Olivieri et al., 2012). Due to their high variability in different *D. melanogaster* strains (McGurk et al., 2021), the three telomeric-associated TE families *Het-A*, *TAHRE*, and *TART* were not considered in our analysis.

#### RNA FISH

RNA FISH was performed using Custom Stellaris® RNA FISH Probes, designed using the Stellaris® RNA FISH Probe Designer (Biosearch Technologies), as previously described (Trcek et al., 2015). RNA FISH Probes were labeled with Quasar670 to detect Cluster 2 (*20A*) sense mRNA sequence. FISH probes were made of 20-nt-long oligo pools, as listed in Table S5. Samples were mounted in Vectashield® media containing DAPI. Fluorescent images were acquired with a Plan-Apochromat 40X/NA1.4 (oil immersion) objective on a Zeiss LSM 780 confocal microscope.

### Quantification and statistical analysis

For all quantification analyses, statistical tests are described in the corresponding figure legends or in the Method details.

## Data Availability

• All raw sequencing data generated in this study have been deposited at the NCBI Gene Expression Omnibus (GEO) repository and are publicly available as of the date of publication. Accession numbers are listed in the key resources table. Original microscopy imaging data have been deposited at Mendeley Data and are publicly available as of the date of publication. The DOI is listed in the Key resources table. • All custom codes used in this study have been deposited at Zenodo and are publicly available as of the date of publication. DOI is listed in the Key resources table. • Any additional information required to reanalyze the data reported in this work paper is available from the Lead Contact upon request.

## Supplemental information

Document S1. Figures S1–S9 and Table S2

Table S1. List of germline piRNA clusters identified in each Drosophila species, related to Figure 1

Table S3. Percentage of uniquely mapping piRNA reads by TE family and piRNA cluster in w1118 ovaries, related to Figure 5

Table S4. Percentage of all mapping piRNA reads by TE family and piRNA cluster (as maximum potential) in w1118 ovaries, related to Figure 5

Table S5. RNA FISH probes, related to Figure 3

Document S2. Article plus supplemental information
