# Supplementary material for: Large Drosophila germline piRNA clusters are evolutionarily labile and dispensable for transposon regulation
Source: Mol Cell. 2021 Oct 7;81(19):3965–3978.e5. doi: 10.1016/j.molcel.2021.07.011 (PMC8516431; doi:10.1016/j.molcel.2021.07.011)
Supplement: Document S1. Figures S1–S9 and Table S2 [file mmc1.pdf]

Molecular Cell, Volume *81*

Supplemental information

Large *Drosophila* germline piRNA clusters are evolutionarily labile and dispensable for transposon regulation

Daniel Gebert, Lena K. Neubert, Catrin Lloyd, Jinghua Gui, Ruth Lehmann, and Felipe Karam Teixeira

Figure S1

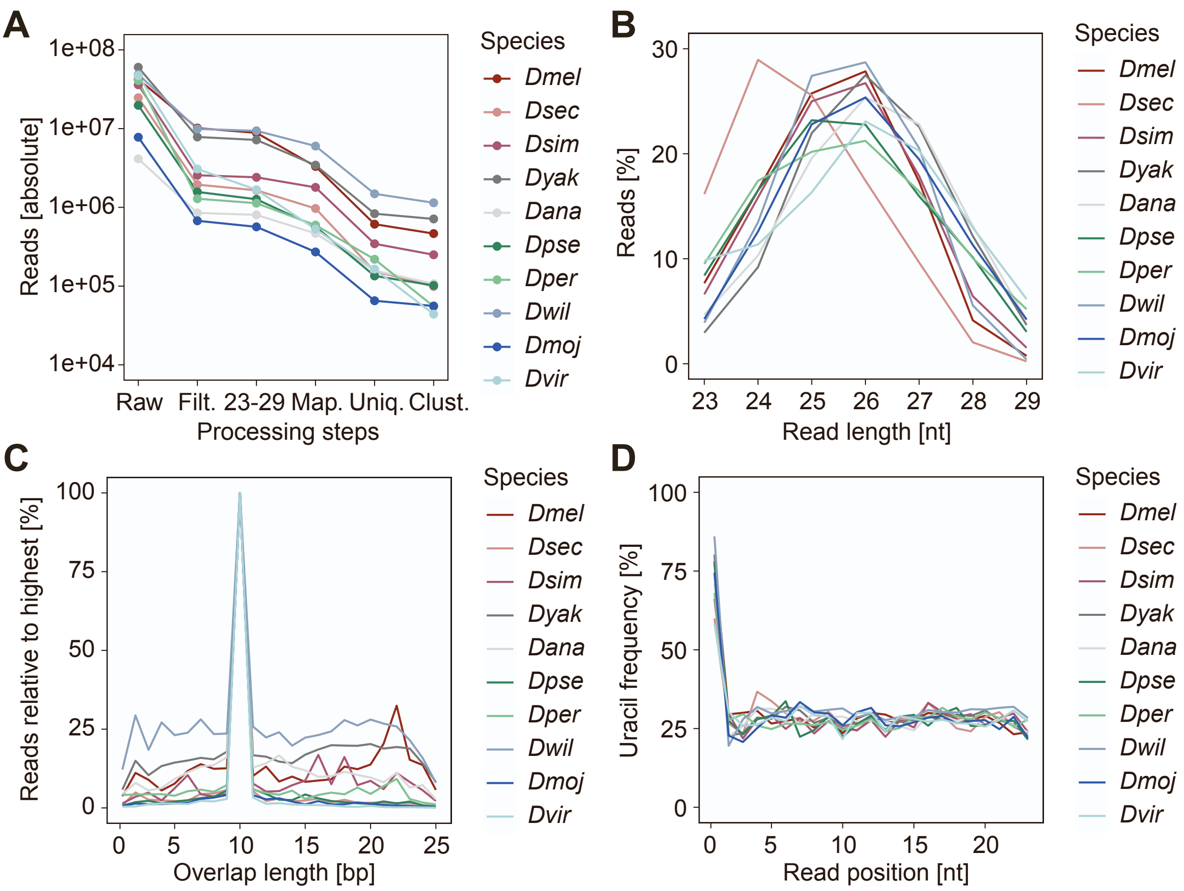
A
Reads [absolute]
1e+08
1e+07
1e+06
1e+05
1e+04
Raw
Filt.
23-29
Map.
Uniq.
Clust.
Processing steps
Species
Dmel
Dsec
Dsim
Dyak
Dana
Dpse
Dper
Dwil
Dmoj
Dvir
B
Reads [%]
30
20
10
0
23
24
25
26
27
28
29
Read length [nt]
Species
Dmel
Dsec
Dsim
Dyak
Dana
Dpse
Dper
Dwil
Dmoj
Dvir
C
Reads relative to highest [%]
100
75
50
25
0
0
5
10
15
20
25
Overlap length [bp]
Species
Dmel
Dsec
Dsim
Dyak
Dana
Dpse
Dper
Dwil
Dmoj
Dvir
D
Uracil frequency [%]
100
75
50
25
0
0
5
10
15
20
Read position [nt]
Species
Dmel
Dsec
Dsim
Dyak
Dana
Dpse
Dper
Dwil
Dmoj
Dvir

Figure S1 (related to Figure 1). Analysis of embryonic small RNA data from 10 *Drosophila* species. (A) Number of reads prior and after filtering. Raw read counts (Raw), non-coding RNA filtering (Filt.), size selection (23-29), genome mapping with all mapped (Map.), genome mapping with unique mapped reads (Uniq.), clustered unique mapped read counts (Clust.). (B) Length distribution of unique mapped piRNA reads. (C) Ping-pong signatures (5' overlaps) of unique mapped piRNA reads. (D) Frequency of uracil bases per position in unique mapped piRNA reads. Dashed line depicts linear regression. r: Pearson correlation coefficient.

Figure S2

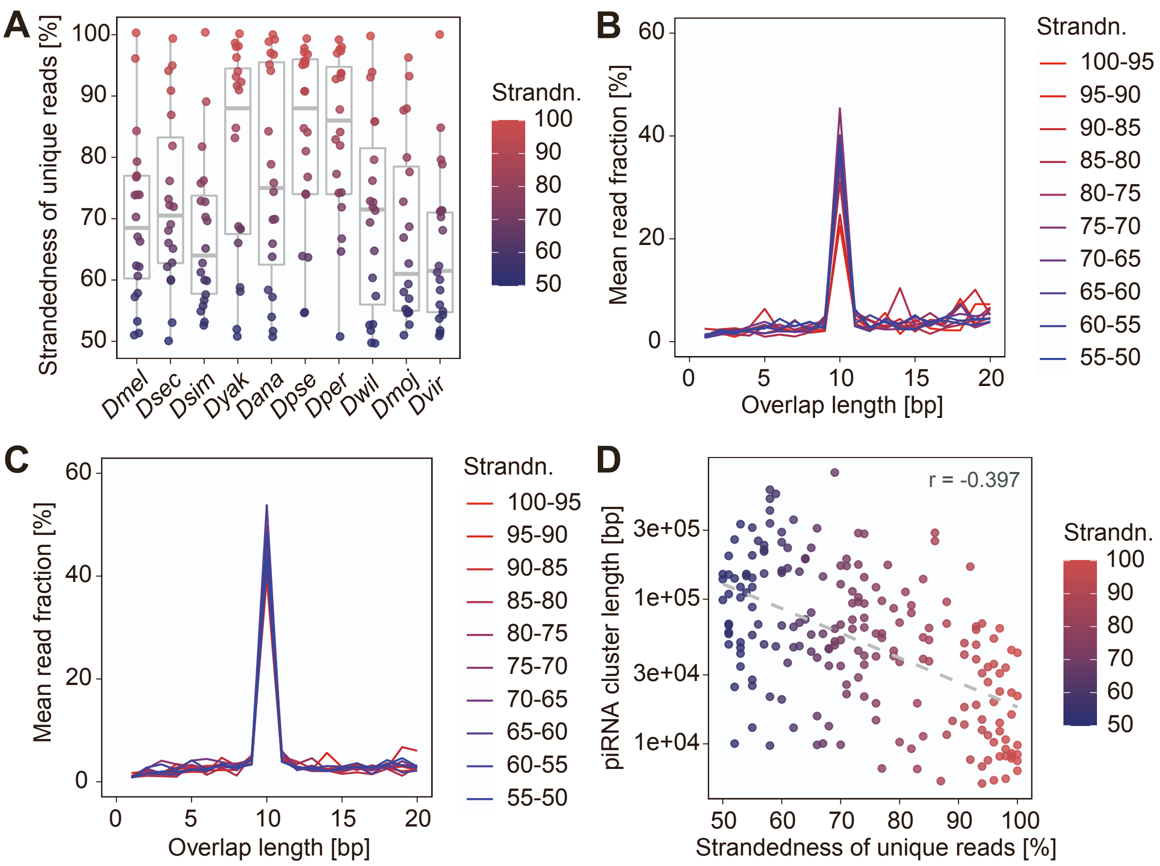
A
Strandedness of unique reads [%]
100
90
80
70
60
50
Dmel
Dsec
Dsim
Dyak
Dana
Dpse
Dper
Dwil
Dmoj
Dvir
Strandn.
100
90
80
70
60
50
B
Mean read fraction [%]
60
40
20
0
0
5
10
15
20
Overlap length [bp]
Strandn.
100-95
95-90
90-85
85-80
80-75
75-70
70-65
65-60
60-55
55-50
C
Mean read fraction [%]
60
40
20
0
0
5
10
15
20
Overlap length [bp]
Strandn.
100-95
95-90
90-85
85-80
80-75
75-70
70-65
65-60
60-55
55-50
D
r = -0.397
piRNA cluster length [bp]
3e+05
1e+05
3e+04
1e+04
50
60
70
80
90
100
Strandedness of unique reads [%]
Strandn.
100
90
80
70
60
50

Figure S2 (related to Figure 1). Strandedness of *Drosophila* germline piRNA clusters. (A) Distribution of strandedness of piRNA clusters in percent of reads on the major strand in each species. Color gradient represents the strandedness in percent of reads on the major strand. (B) Ping-pong signatures (5' overlaps) of unique mapped piRNA cluster reads in different ranges of strandedness. (C) Ping-pong signatures (5' overlaps) of all mapped piRNA cluster reads in different ranges of strandedness. (D) Correlation of piRNA cluster length and strandedness.

Figure S3

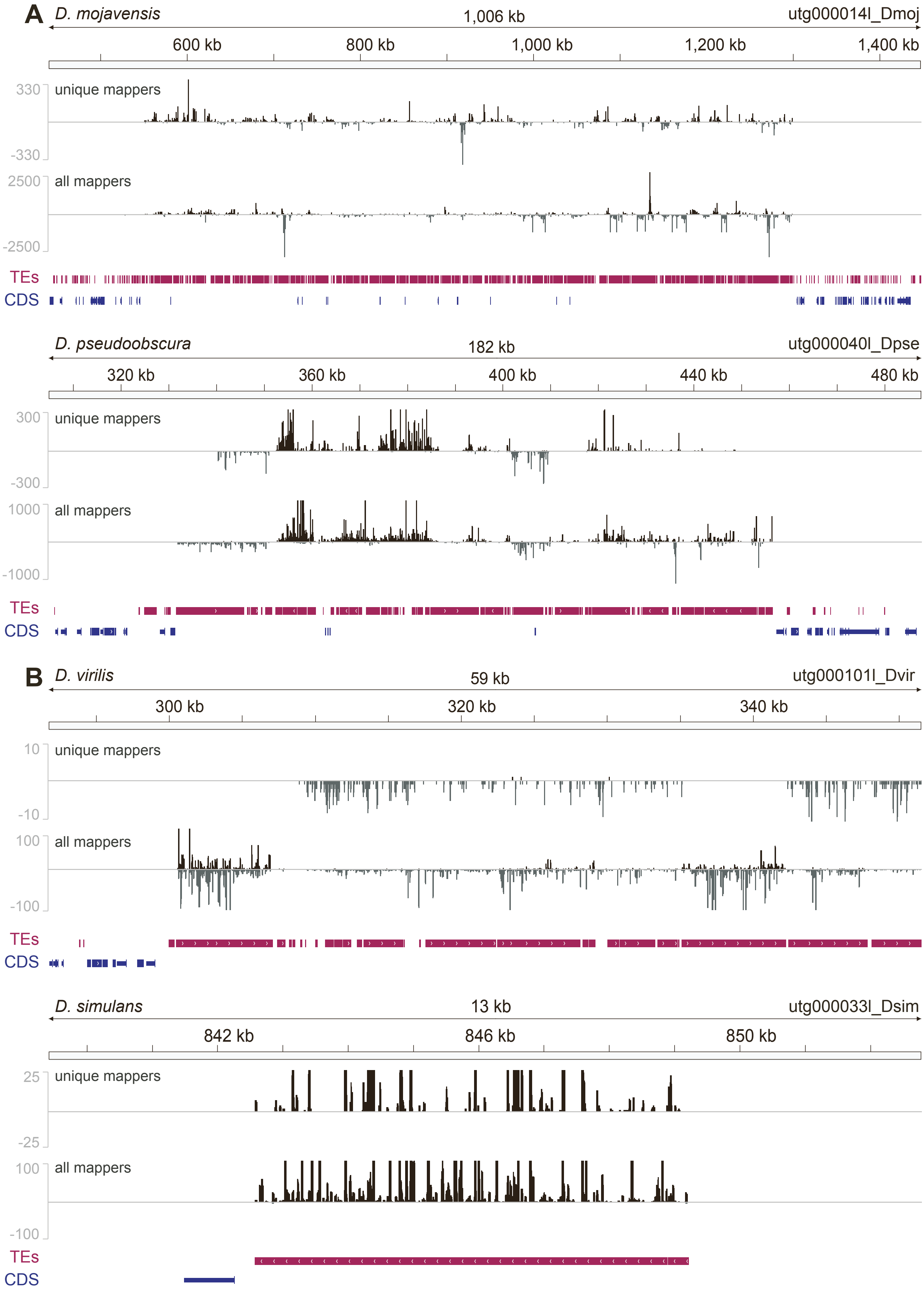

A
D. mojavensis
1,006 kb
utg000014l_Dmoj
600 kb
800 kb
1,000 kb
1,200 kb
1,400 kb
330
unique mappers
-330
2500
all mappers
-2500
TEs
CDS
D. pseudoobscura
182 kb
utg000040l_Dpse
320 kb
360 kb
400 kb
440 kb
480 kb
300
unique mappers
-300
1000
all mappers
-1000
TEs
CDS
B
D. virilis
59 kb
utg000101l_Dvir
300 kb
320 kb
340 kb
10
unique mappers
-10
100
all mappers
-100
TEs
CDS
D. simulans
13 kb
utg000033l_Dsim
842 kb
846 kb
850 kb
25
unique mappers
-25
100
all mappers
-100
TEs
CDS

Figure S3. Examples of dual-strand and uni-strand germline piRNA clusters in the *Drosophila* genus (related to Figure 1). (A) Dual-strand piRNA clusters in *D. mojavensis* and *D. pseudoobscura*. (B) Uni-strand piRNA clusters in *D. virilis* and *D. simulans*.

Figure S4

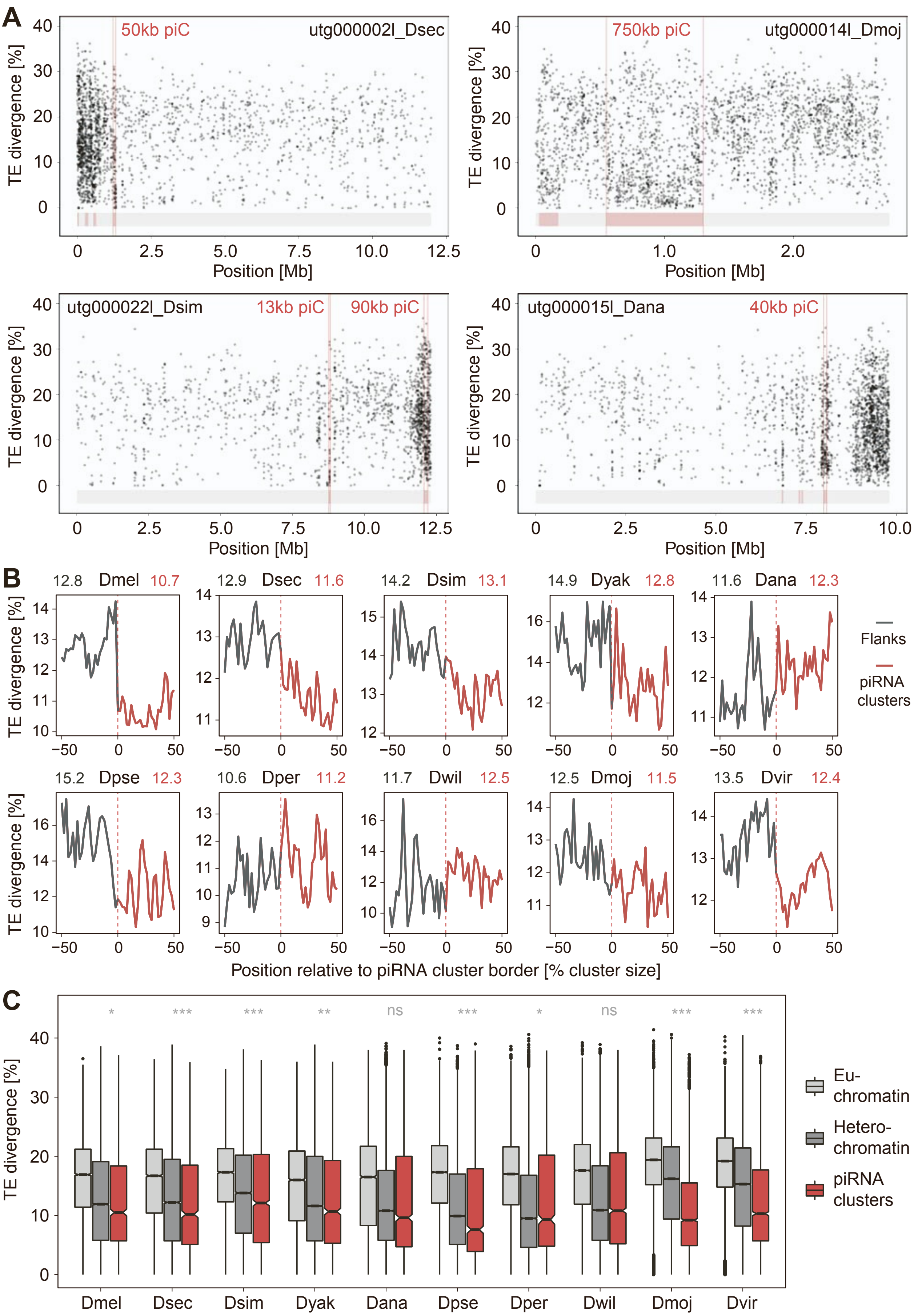

A
50kb piC
utg000002l_Dsec
750kb piC
utg000014l_Dmoj
utg000022l_Dsim
13kb piC
90kb piC
utg000015l_Dana
40kb piC
TE divergence [%]
Position [Mb]
B
12.8 Dmel 10.7
12.9 Dsec 11.6
14.2 Dsim 13.1
14.9 Dyak 12.8
11.6 Dana 12.3
15.2 Dpse 12.3
10.6 Dper 11.2
11.7 Dwil 12.5
12.5 Dmoj 11.5
13.5 Dvir 12.4
Flanks
piRNA clusters
Position relative to piRNA cluster border [% cluster size]
C
* *** *** ** ns *** * ns *** ***
Dmel Dsec Dsim Dyak Dana Dpse Dper Dwil Dmoj Dvir
Eu-chromatin
Hetero-chromatin
piRNA clusters

Figure S4 (related to Figure 2). Analysis of sequence divergence of transposons in the *Drosophila* genus. (A) Percentage of sequence divergence from consensus for individual TE copies across *Drosophila* chromosomes/contigs. piRNA clusters are highlighted in red. (B) Average TE sequence divergence at the borders (dashed line) of the top largest germline piRNA clusters of each species, including halves (50% length) of internal cluster sequences and flanking regions of corresponding lengths (-50%). Means of TE sequence divergence of flanking regions and piRNA clusters are shown for each species above plots. (C) Distributions of sequence divergence from consensus of TE copies located in piRNA clusters, heterochromatin, and euchromatin in each *Drosophila* species. Statistical significance in comparisons between TE divergence distributions in heterochromatin and piRNA clusters was determined using a Mann-Whitney U test (Wilcoxon rank sum test). ns: $p>0.05$; *$p<0.05$; **: $p<0.01$; ***: $p<0.001$.

Figure S5

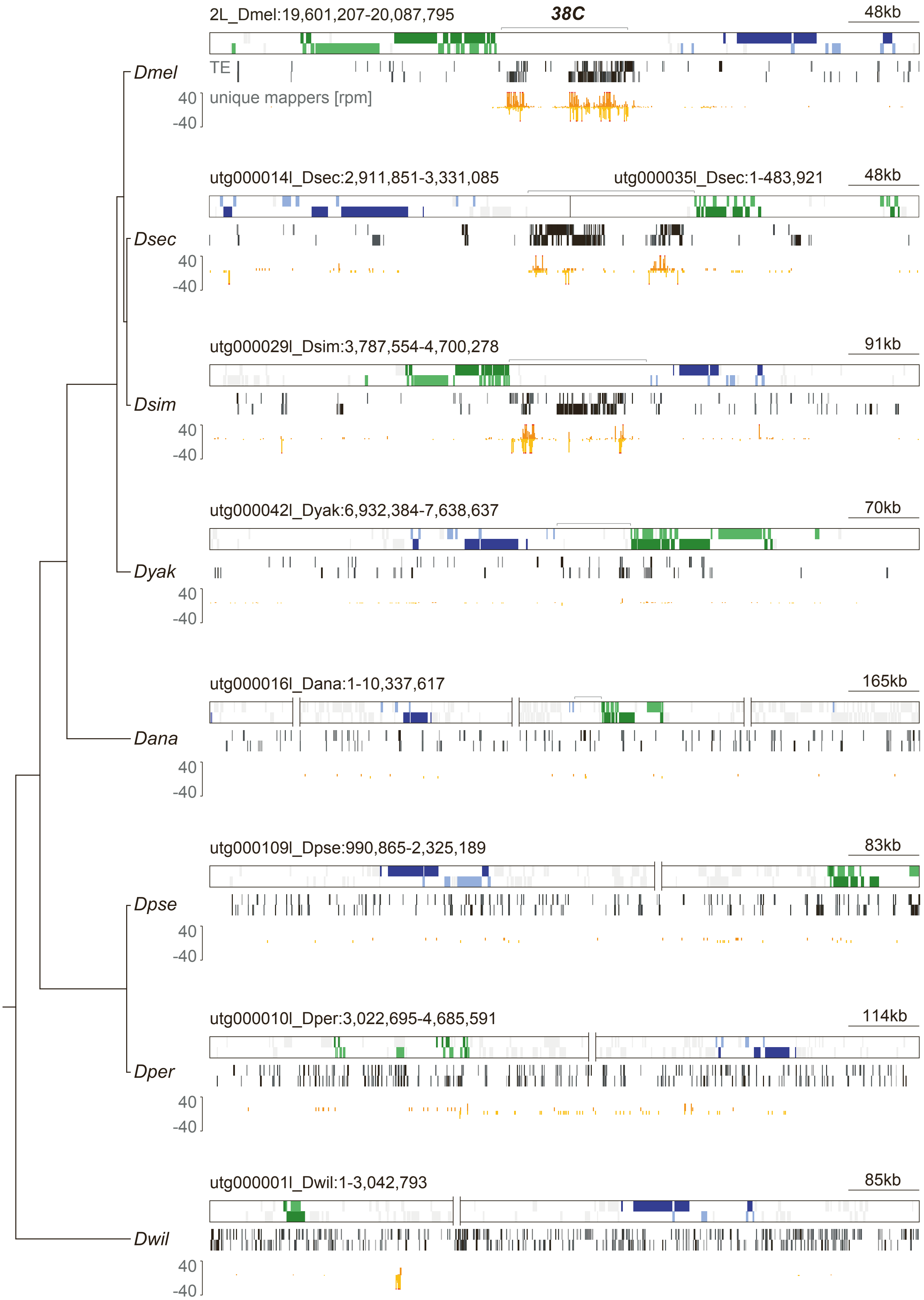

2L_Dmel:19,601,207-20,087,795
38C
48kb
Dmel
TE
unique mappers [rpm]
40
-40
utg000014l_Dsec:2,911,851-3,331,085
utg000035l_Dsec:1-483,921
48kb
Dsec
utg000029l_Dsim:3,787,554-4,700,278
91kb
Dsim
utg000042l_Dyak:6,932,384-7,638,637
70kb
Dyak
utg000016l_Dana:1-10,337,617
165kb
Dana
utg000109l_Dpse:990,865-2,325,189
83kb
Dpse
utg000010l_Dper:3,022,695-4,685,591
114kb
Dper
utg000001l_Dwil:1-3,042,793
85kb
Dwil

Figure S5 (related to Figure 2). Evolution of *D. melanogaster* piRNA cluster *38C* across *Drosophila* genus. Top track: upstream flanking genes of *D. melanogaster* piRNA cluster *38C* on plus strand (dark green), upstream flanking genes on minus strand (light green), downstream flanking genes on plus strand (dark blue), downstream flanking genes on minus strand (light blue). Split in the *D. sechellia* (Dsec) represents a discontinuity in the nanopore long-read-based genome assembly (Miller et al., 2018) that is continuous in the original assembly (Drosophila 12 Genomes et al., 2007). Middle track: TE insertions on plus and minus strand (black). Bottom track: piRNA coverage on plus and minus strand in reads per million.

Figure S6

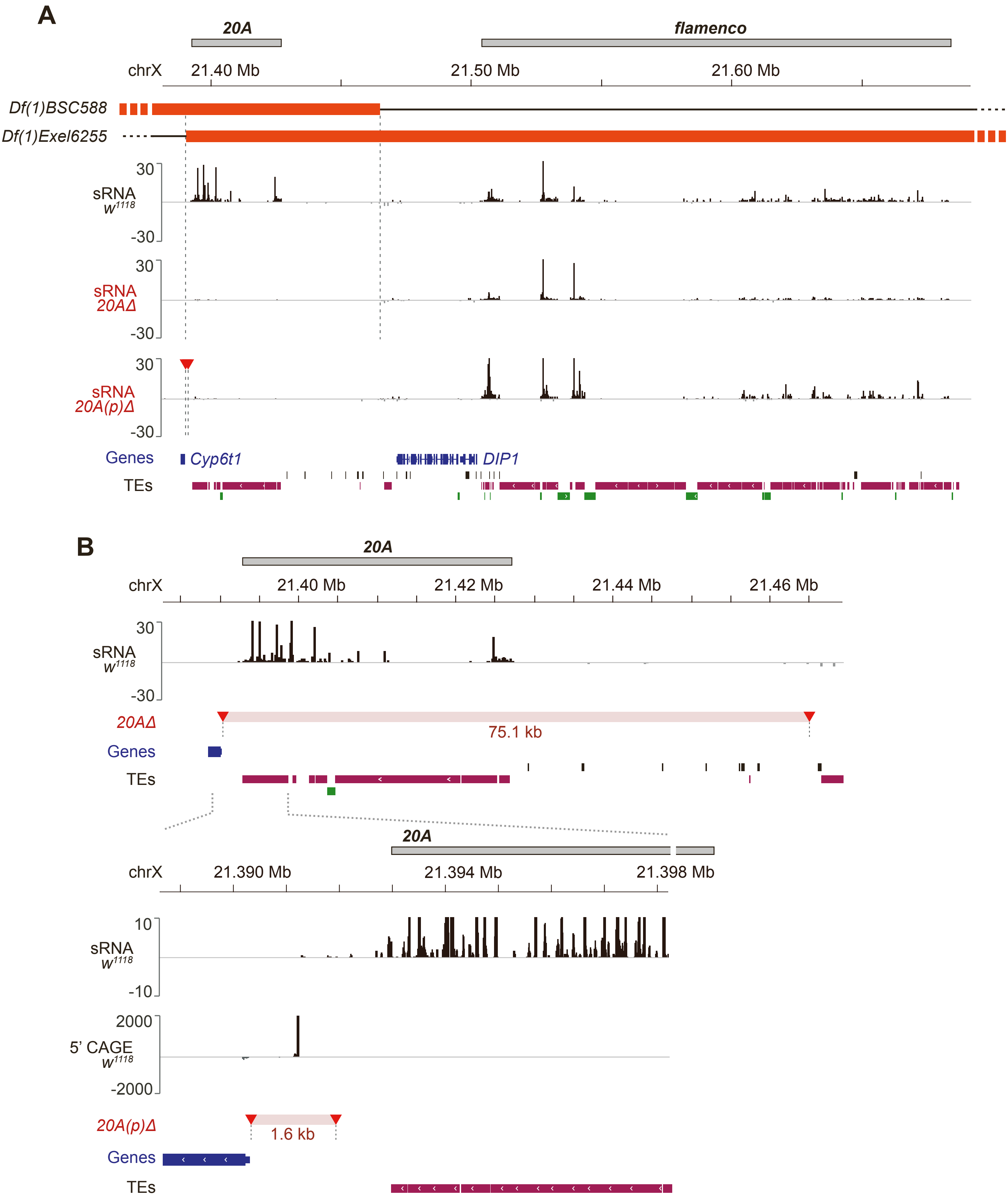

A
20A
flamenco
chrX
21.40 Mb
21.50 Mb
21.60 Mb
Df(1)BSC588
Df(1)Exel6255
30
sRNA w1118
-30
30
sRNA 20AΔ
-30
30
sRNA 20A(p)Δ
-30
Genes
Cyp6t1
DIP1
TEs
B
20A
chrX
21.40 Mb
21.42 Mb
21.44 Mb
21.46 Mb
30
sRNA w1118
-30
20AΔ
75.1 kb
Genes
TEs
20A
chrX
21.390 Mb
21.394 Mb
21.398 Mb
10
sRNA w1118
-10
2000
5' CAGE w1118
-2000
20A(p)Δ
1.6 kb
Genes
TEs

Figure S6 (related to Figure 3). Deletions of the germline piRNA cluster *20A* in *D. melanogaster*. (A) Genomic browser view of neighboring piRNA clusters *20A* and *flamenco* on *D. melanogaster* X chromosome. Small RNA-seq uniquely mapping read tracks for *w1118*, *20A*Δ*,* and *20A(p)*Δ. FRT sites are represented by red arrows. Large chromosomal deletions *Df(1)BSC588* and *Df(1)Exel6255* are represented at the top (orange boxes represent deleted DNA). Annotation is at the bottom: genes (blue), DNA transposons (black), LTR retrotransposons (purple), and non-LTR retrotransposons (green). (B) Zoom-in views for *20A*Δ and *20A(p)*Δ. Annotation as in (A).

Figure S7

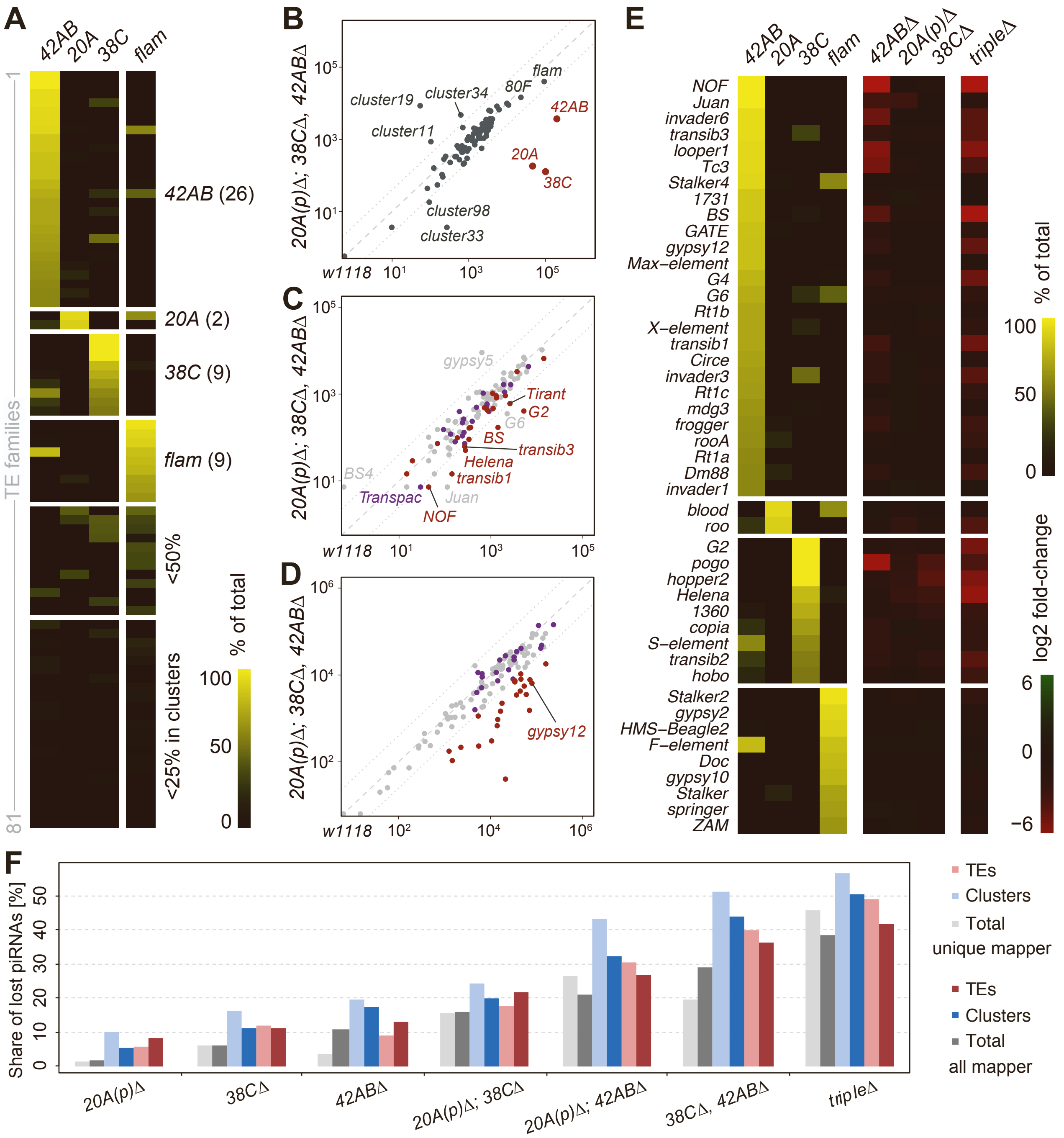

A
42AB
20A
38C
flam
TE families
1
81
42AB (26)
20A (2)
38C (9)
flam (9)
<50%
<25% in clusters
% of total
100
50
0
B
20A(p)Δ; 38CΔ, 42ABΔ
w1118
flam
80F
cluster19
cluster34
cluster11
42AB
20A
38C
cluster98
cluster33
C
gypsy5
Tirant
G2
G6
BS
transib3
Helena
transib1
BS4
Transpac
Juan
NOF
D
gypsy12
E
42ABΔ
20A(p)Δ
38CΔ
tripleΔ
NOF
Juan
invader6
transib3
looper1
Tc3
Stalker4
1731
BS
GATE
gypsy12
Max–element
G4
G6
Rt1b
X–element
transib1
Circe
invader3
Rt1c
mdg3
frogger
rooA
Rt1a
Dm88
invader1
blood
roo
G2
pogo
hopper2
Helena
1360
copia
S–element
transib2
hobo
Stalker2
gypsy2
HMS–Beagle2
F–element
Doc
gypsy10
Stalker
springer
ZAM
% of total
log2 fold-change
6
0
−6
F
Share of lost piRNAs [%]
0
10
20
30
40
50
20A(p)Δ
38CΔ
42ABΔ
20A(p)Δ; 38CΔ
20A(p)Δ; 42ABΔ
38CΔ, 42ABΔ
tripleΔ
TEs
Clusters
Total
unique mapper
TEs
Clusters
Total
all mapper

Figure S7 (related to Figure 5). Effects of piRNA cluster deletions on piRNA accumulation. (A) Distribution of piRNA reads (percentage of all mapped reads) matching each TE family and piRNA clusters (*42AB*, *20A*, *38C*, *flamenco* (*flam*), and all the remaining clusters) in w^{1118} ovaries. (B) Scatterplot showing the accumulation of uniquely mapping piRNA reads matching piRNA clusters in triple mutant ovaries in comparison to w^{1118} control. Dashed line represents perfect x=y correlation. Dotted lines delineate 4-fold change. (C) Uniquely mapping piRNA reads on dispersed TE copies located outside piRNA clusters in triple mutant ovaries in comparison to w^{1118} control. TE families with >75% loss of piRNAs in triple mutant ovaries compared to w^{1118} control are shown in red. TE families with reactivation in germline knockdown of PIWI proteins (*piwi, aub*, or *ago3*; Senti et al., 2015) is shown in purple. All other families are displayed in gray. (D) TE-matching piRNA reads (all mappers) per TE family in triple mutant ovaries in comparison to w^{1118} control. Same coloring as in C. *Gypsy12* family falls in two categories: >75% loss of piRNAs in triple mutant ovaries and reactivation in germline knockdowns of PIWI proteins (Senti et al., 2015). (E) Distribution of piRNA reads (percentage of all mapped reads) matching each TE family and piRNA clusters (*42AB*, *20A*, *38C*, and *flam*) in w^{1118} ovaries (yellow gradient) and the $\log_2$ fold change of TE-matching piRNA reads (all mapped reads) for each family in mutant ovaries in comparison to w^{1118} control (red-green gradient). Correlation coefficients (r) between percentage of mappers in *w1118* and $\log_2$ fold change in mutants: -0.369 (*42AB*Δ, p=0.0116), -0.274 (*20A(p)*Δ, p=0.0649), and -0.847 (*38C*Δ, p<0.0001). (F) Percentage of loss of total, clustered and TE-matching piRNA reads (all mapped and unique mapped reads) in piRNA cluster mutant ovaries when compared to w^{1118} control.

Figure S8

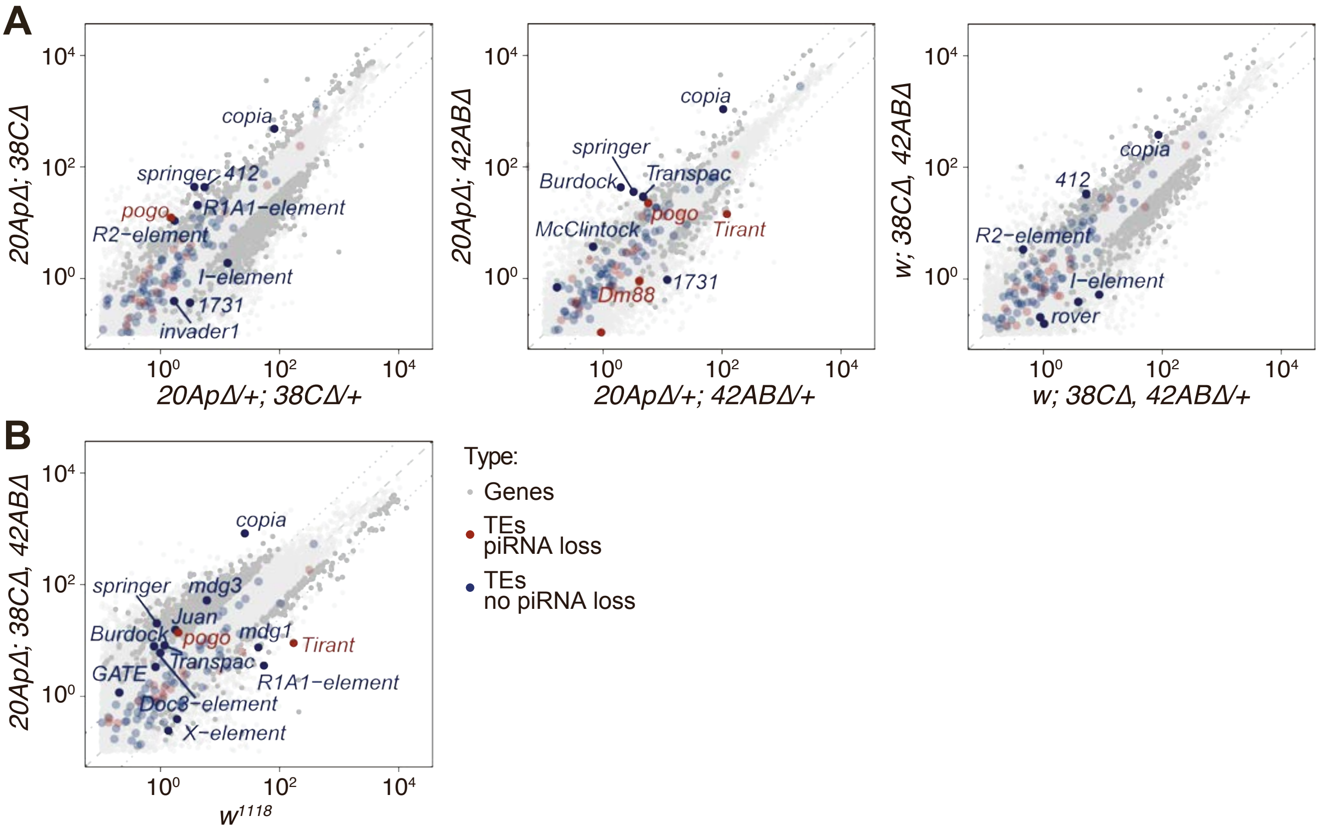
A
copia
springer
412
pogo
R1A1−element
R2−element
I−element
1731
invader1
20ApΔ; 38CΔ
20ApΔ/+; 38CΔ/+
springer
Burdock
Transpac
McClintock
pogo
Tirant
Dm88
1731
20ApΔ; 42ABΔ
20ApΔ/+; 42ABΔ/+
412
R2−element
I−element
rover
w; 38CΔ, 42ABΔ
w; 38CΔ, 42ABΔ/+
B
springer
mdg3
Juan
Burdock
pogo
mdg1
Tirant
Transpac
GATE
R1A1−element
Doc3−element
X−element
20ApΔ; 38CΔ, 42ABΔ
w1118
Type:
Genes
TEs
piRNA loss
TEs
no piRNA loss

Figure S8 (related to Figure 6). Impact of piRNA cluster deletions on TE expression. (A) Scatterplot showing the expression of genes (gray dots; dark gray indicates differentially expressed genes; q-value>0.01; 0.5<log2<-0.5), transposons with >75% piRNA loss in triple mutants (red dots), and all other transposon families (blue dots) as measured by RNA-seq analysis (expressed in fragments per kilobase per million fragments, FPKM, $\log_{10}$), in *20A(p)Δ/+; 38CΔ/+* heterozygous vs. *20A(p)Δ; 38CΔ* homozygous mutant ovaries, *20A(p)Δ/+; 42ABΔ/+* heterozygous vs. *20A(p)Δ; 42ABΔ* homozygous mutant ovaries, *38CΔ/+, 42ABΔ/+* heterozygous vs. *38CΔ, 42ABΔ* homozygous mutant ovaries. (B) w^{1118} vs. *20A(p)Δ; 38CΔ, 42ABΔ* homozygous mutant ovaries. Dashed line represents perfect x=y correlation. Dotted lines delineate 4-fold change.

Figure S9

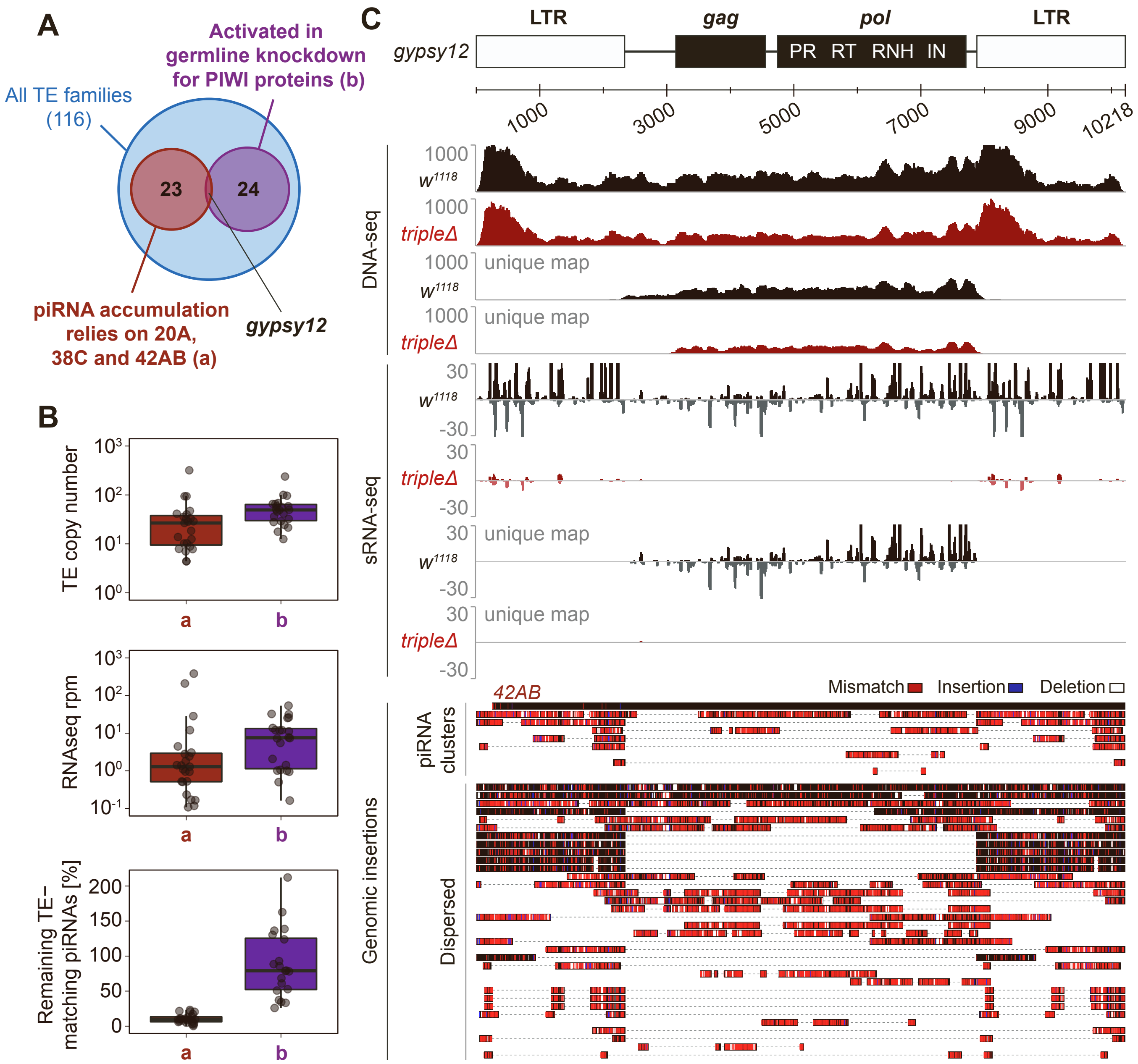
A
Activated in germline knockdown for PIWI proteins (b)
All TE families (116)
23
24
piRNA accumulation relies on 20A, 38C and 42AB (a)
gypsy12
B
TE copy number
RNAseq rpm
Remaining TE-matching piRNAs [%]
a
b
C
gypsy12
LTR
gag
pol
PR RT RNH IN
1000
3000
5000
7000
9000
10218
DNA-seq
sRNA-seq
w1118
tripleΔ
unique map
42AB
Mismatch
Insertion
Deletion
Genomic insertions
piRNA clusters
Dispersed

Figure S9 (related to Figure 6). Comparison of TEs with substantial piRNA loss in piRNA cluster deletions and reactivated TEs in germline piRNA pathway mutants. (A) Venn diagram of all TE families, TE families with >75% piRNA loss in the triple mutant ('a') and TE families with reactivation in germline piRNA mutants ('b'). (B) Top: Distribution of genomic copy number as measured by DNA-seq data analysis (expressed in read bps per TE bps divided by genomic coverage depth, $\log_{10}$) of TE families in groups 'a' and 'b'. Mid: Distribution of expression as measured by RNA-seq analysis (expressed in fragments per kilobase per million fragments, FPKM, $\log_{10}$). Bottom: Distribution of the percentage of remaining piRNAs (all mappers) in triple mutant ovaries in comparison to w^{1118} control. (C) Analysis of the *gypsy12* family. Diagram of the consensus *gypsy12* retrotransposon element (10,218bp-long) is shown at the top: white boxes, long terminal repeats (LTR); black box, *gag* and *pol* coding sequences (PR: protease; RT: reverse transcriptase; RNH: ribonuclease H; IN: integrase). Top panels: Density plots for all and uniquely mapping DNA-seq reads (DNA-seq) in w^{1118} and triple mutants. Middle panels: Density plots for all and uniquely mapping small RNA-seq reads (sRNA-seq) in w^{1118} and triple mutants. Bottom panels: *gypsy12* insertions in the *D. melanogaster* genome (dm6), grouped by genomic location (within piRNA clusters or dispersed copies outside clusters). Near full-length insertion in piRNA cluster *42AB* is shown at the top.

Table S2 (related to Figure 3). Coordinates of FRT sites used for piRNA cluster deletions.

Chromosome	Position	Name	Stock number (insertion name)
chr2L	20104769	38CDf1_FRT1	Kyoto# 124205 (P{RS3}CB-6748-3)
chr2L	20243057	38CDf1_FRT2	Kyoto# 126282 (P{RS5}5-SZ-4007)
chr2R	2159264	42ABDf1_FRT1	Exelixis# e04172 (PBac{RB}e04172)
chr2R	2389366	42ABDf1_FRT2	Exelixis# d00877 (P{XP}d00877)
chrX	21390230	Cl2pD_FRT1	Exelixis# d03497 (P{XP}d03497)
chrX	21391839	Cl2pD_FRT2	Exelixis# f02310 (PBac{WH}f02310)
